# A Novel SP1/SP3 Dependent Intronic Enhancer Governing Transcription of the UCP3 Gene in Brown Adipocytes

**DOI:** 10.1371/journal.pone.0083426

**Published:** 2013-12-31

**Authors:** Christoph Hoffmann, Anika Zimmermann, Anke Hinney, Anna-Lena Volckmar, Harry W. Jarrett, Tobias Fromme, Martin Klingenspor

**Affiliations:** 1 Molecular Nutritional Medicine, Else Kröner-Fresenius Center & Research Center for Nutrition and Food Sciences, Technische Universität München, Freising-Weihenstephan, Germany; 2 Department of Child and Adolescent Psychiatry, Psychosomatics and Psychotherapy, Universitätsklinikum Essen, University of Duisburg-Essen, Essen, Germany; 3 Department of Chemistry, University of Texas at San Antonio, San Antonio, Texas, United States of America; The Hong Kong Polytechnic University, Hong Kong

## Abstract

Uncoupling protein (UCP) 3 is a mitochondrial inner membrane protein implicated in lipid handling and metabolism of reactive oxygen species. Its transcription is mainly regulated by peroxisome proliferator-activated receptors (PPAR), a family of nuclear hormone receptors. Employing bandshift assays, RNA interference and reporter gene assays we examine an intronic region in the UCP3 gene harboring a *cis*-element essential for expression in brown adipocytes. We demonstrate binding of SP1 and SP3 to this element which is adjacent to a direct repeat 1 element mediating activation of UCP3 expression by PPARγ agonists. Transactivation mediated by these elements is interdependent and indispensable for UCP3 expression. Systematic deletion uncovered a third binding element, a putative NF1 site, in close proximity to the SP1/3 and PPARγ binding elements. Data mining demonstrated binding of MyoD and Myogenin to this third element in C2C12 cells, and, furthermore, revealed recruitment of p300. Taken together, this intronic region is the main enhancer driving UCP3 expression with SP1/3 and PPARγ as the core factors required for expression.

## Introduction

Uncoupling protein (UCP) 3 and its two paralogues, UCP1 and UCP2, belong to the mitochondrial anion transporter superfamily. All are located in the mitochondrial inner membrane, but differ significantly in tissue distribution. While UCP1 is restricted to brown adipose tissue (BAT) and UCP2 is expressed almost ubiquitously, UCP3 can only be found in BAT, skeletal muscle (SKTM) and heart [1], [2].

It is commonly accepted that UCP1, directly or indirectly, allows protons to pass the mitochondrial inner membrane [3] enabling fuel combustion to run at maximal capacity for the purpose of thermogenesis. None of the other UCPs directly contributes to thermogenesis [4]. Their uncoupling activity, however, may be of importance for other processes. Both UCP2 and UCP3 may function as valves preventing an excessive proton gradient which would lead to increased generation of ROS [5]. Additionally, they have been proposed to play a role in calcium transport [6] and glucose sensitivity [7].

UCP3 has also been suggested to transport lipid radicals, fatty acids, and pyruvate. The export of lipid radicals could prevent damage of mitochondrial DNA and matrix enzymes [8], the export of fatty acids may be part of a mechanism preventing coenzyme A shortage in the matrix [9] and prevent lipid-induced mitochondrial damage [10], while the transport of pyruvate would ensure equilibrium between glycolysis and oxidative phosphorylation [11].

An involvement in fatty acid metabolism for UCP3 is supported by its physiological regulation. UCP3 expression is increased in fasting [12], [13], exercise [14], [15], high fat feeding [16], [17] and cold exposure [18], [19]. All these conditions are accompanied by increased lipid levels in plasma which corresponds with the observation of increased UCP3 expression in response to direct lipid infusion [20].

On a molecular level, the peroxisome proliferator activated receptors (PPARs) play a key role in regulation of UCP3 expression [21]. Their binding site is thought to be a Direct Repeat 1 (DR1) site within the promoter region. It is unclear which PPAR isoforms confers induction of expression in response to different challenges and in different tissues. For BAT, the most important PPAR seems to be PPARγ. PPARγ ligands activate UCP3 expression in animal models [22] and cell culture [23]. Furthermore, UCP3 in BAT is induced by PPARα agonists, the effect being additive to the PPARγ effect [24].

While PPARα and PPARγ show higher expression in BAT as compared to SKTM, PPARδ expression seems to be comparable in both tissues. PPARδ agonists increase the abundance of UCP3 protein in SKTM [25] and L6 myoblasts. Taken together, for SKTM PPARδ and PPARγ seem to be regulators for UCP3 transcription, while in BAT PPARγ and PPARα dominate [24], [26].

Recently, we discovered a naturally occurring mutation (intervening sequence (IVS)1+1505G→A) in the Djungarian hamster (*Phodopus sungorus)* which completely abolishes UCP3 expression in BAT in vivo, but has only minor effects on SKTM expression. BAT specific absence of UCP3 in this model leads to increased body weight, impaired cold tolerance and reduction of mRNA abundance for several enzymes involved in macronutrient metabolism [27], [28]. A reporter gene construct harboring both UCP3 promoter and first intron responds to PPARγ agonists in the hibernoma 1b (HIB1b) brown fat cell line and immortalized brown preadipocytes (iBPAs), but only poorly in the muscle cell lines C2C12 and L6. The induction is abolished by the IVS1+1505G→A mutation. Subsequently, the presence of a second DR1 element binding PPARγ/RXRα less than 100 bp upstream of the IVS1+1505G element was reported [29].

We scanned the first intron of the UCP3 gene for regions harboring cis-elements, searched for transcription factors binding to candidate regions, and dissected the relative contribution of the regulatory regions to UCP3 gene expression. Our goal was to identify the proteins binding to the IVS1+1505G element and inspect the interplay between IVS1+1505G and the DR1 elements. Furthermore we used deletion constructs and data mining to search for other elements harbored in the first intron of UCP3 and influence its expression. Taken together, our study characterizes a novel complex regulatory region: The UCP3 enhancer. Binding sites for SP1/3 and PPARγ/RXRα form the core of this enhancer, and are interdependent and indispensable for expression of UCP3. A PPAR/RXR binding element in the proximal promoter is of lesser importance and depends on presence of both intronic elements. The enhancer contains at least one more element, binding MyoD and Myogenin in SKTM, and is able to recruit p300, a histone acetylase.

## Materials and Methods

### Materials

All basic chemicals, unless otherwise stated, were purchased at Carl Roth (Karlsruhe, Germany). Plastic- and cell culture ware was purchased from Sarstedt (Nümbrecht, Germany). Enzymes were manufactured by Fermentas (St. Leon-Rot, Germany). Sequencing and oligonucleotide synthesis was carried out by Eurofins MWG Operon (Ebersberg, Germany). Deletion and QuickChange primers can be found in Table S1, miRNA sequences and combinations in Table S2 and , PCR primers for amplification of the miRNA cassette in Table S4, shRNA sequences in Table S5, EMSA probes and competitors in Table S6 and sequencing primers in Table S7. Oligonucleotides for generating the overexpression constructs can be found in Table S8.

### Vector Construction

Generation of the UCP3 reporter gene vectors is described in [27]. Deletion constructs were generated by PCR using Phusion DNA polymerase (Finnzymes, Vantaa Finland) according to manufacturer’s protocol. Primers were designed to flank the region to be deleted, amplifying the rest of the vector. PCR products were phosphorylated, recircularized and deletions were validated by restriction analysis. For all generated constructs deleted region, promoter, intronic enhancer and luciferase open reading frame were sequenced to exclude introduction of mutations. To disrupt the two DR1 sites, the QuickChangeII mutagenesis kit (Agilent, Santa Clara, California, USA) was used to either insert an EcoRV recognition site (promoter) or XhoI recognition site (intron), respectively. In all generated constructs we sequenced promoter, intronic enhancer and luciferase.

miRNA sequences were generated using the BlockIt miRNA design tool (Invitrogen, Carlsbad, California, USA) and annealed and inserted into pcDNA6.2 emGFP miR (Invitrogen) Vector according to the manufacturer’s protocol. For each target, two miRNAs were cloned and concatemerised. The miRNA combinations used can be found in Table S3. emGFP-miRNA cassettes were amplified using the primers by Phusion polymerase and inserted into pJet 1.2 blunt (Fermentas). Constructs were then sequenced from the pJet fw sequencing primer. The cassette was excised using Eco31I, generating ends compatible with BamHI and XhoI. The fragment was then ligated into pMXs-IRES-Puro (Cell Biolabs, San Diego, California, USA) that was linearised with BamHI and XhoI.

Overexpression constructs were generated by amplifying the full length transcript from BAT cDNA with Phusion Polymerase. Using primers containing restriction sites, the PCR products where then inserted in pMXs EF1 PGK BSD as described for the miRNA cassettes. The Ty1 epitope Tag sequence was annealed from 2 complementary oligonucleotides and inserted into pMXs before inserting the cDNAs to generate N-terminal fusions.

### Cell Culture

Platinum E cells were cultured in DMEM high glucose (Sigma, St. Louis, Missouri, USA) supplemented with 10% FBS superior (BioChrom, Berlin, Germany) and 20 µg/ml Gentamycin (BioChrom). At 80–100% confluency, cells were split 1∶7 using typsin/EDTA solution (BioChrom). Every four weeks cells were selected by addition of 10 µg/ml Blasticidin and 1 µg/ml Puromycin (both Invivogen, San Diego, California) for two passages to ensure expression of viral packaging genes. Hib1b-cells [30] were cultured in DMEM:F12 (Invitrogen) supplemented with glucose to a concentration of 6 g/l. At 80–100% confluency, cells were split 1∶7. For transient transfection, cells were split the day before transfection 1∶3 to ensure cells were in their logarithmic growth phase the day of transfection. For differentiation medium was changed to differentiation medium (7% FBS, 20 µM human insulin (Sigma), 1 nM triiodothyronin (Sigma), 93% supplemented DMEM:F12, 20 µg/ml Gentamycin), replacing fresh medium every other day. For induction, differentiation medium was supplemented with 3-isobutyl-1-methylxanthine (500 µM), dexamethasone (2 µg/ml) and indomethacin (65 µM) (induction medium). Immortalised brown preadipocytes (iBPA) [31] were essentially cultured like Hib1b cells except for using DMEM high glucose instead of DMEM:F12. Serum concentration was kept at 10% during the whole differentiation. Plantinum E cells were purchased from Cell Biolabs. HIB1b and iBPA cells were kindly provided by Bruce Spiegelman (Dana-Farber Cancer Institute, Harvard Medical School) and Patrick Seale (Institute for Diabetes, Obesity and Metabolism, Philadelphia), respectively. A second, fresh batch of iBPA cells was kindly provided by Ana Kilic and the lab of Alexander Pfeifer (Institute of Pharmacology and Toxicology, University of Bonn).

### Luciferase Assay

Hib1b and immortalized brown preadipocytes (iBPA) were seeded onto 96 well plates and transfected 3 hours later using Lipofectamin LTX (Invitrogen) (0.25 µl per well) or Nucleofector 96 (Amaxa, Gaithersburg, Maryland, USA) (Soution SE, CM137). Each well received reporter gene construct (pGL3 (Promega, Wisconsin, USA), gaussia luciferase) and a transfection control (cmv driven photinus luciferase, pGL3) along with control- or RNAi-vector. 16 hours later, medium was changed to either differentiation medium (including agonists where indicated, all non-RNAi experiments) or induction medium (iBPA, HIB1b for RNAi experiments). Cells were lysed either 48 hours after transfection for all non-RNAi experiments, 96 h after for shRNA experiments and 120 h after for miRNA experiments by the addition of 25 µl 1× passive lysis buffer (Promega) per well. Luciferase assay was carried out using the Promega dual luciferase assay kit by a Tecan Infinite M200 (Männedorf, Switzerland) plate reader in white 96 well plates. Reporter gene activity was normalized to the transfection control. Experiments were inter-day normalized.

### Viral Transduction

10^6^ Platinum E cells were seeded per 6 cm dish and transfected 3 hours later using the calcium phosphate method. 16 h later, cells received fresh medium and further 24 h later supernatants were harvested and filtered sterile through 0.22 µm filters and stored aliquoted at −80°C. Retroviral titers were measured by linear dilution, infection, selection and counting colonies. Hib1b cells were seeded at in a 12 well plates, seeded and infected 3 hours later at a MOI of 0,3. 24 h later, medium was removed and fresh medium containing 1.5 µg/ml puromycin was added. 48 h later cells were split into fresh medium containing puromycin and infection was assayed by GFP fluorescence. After further 48 h of selection cells were split for transfection.

### EMSA

All steps, unlike otherwise stated, were carried out at 4°C or on ice. HIB1b cells were split onto 15 cm dishes and cultured until confluency. Medium was replaced by differentiation medium and differentiated for 8 days. Medium was changed every other day. Double stranded oligonucleotides and nuclear exctracts were prepared as described in [27]. For EMSA, 3–5 µg nuclear extract, Buffer (10x: 40% (vol/vol) glycerol, 10 mM MgCl_2_, 5 mM EDTA, 5 mM DTT, 500 mM, NaCl, 100 mM TrisHCl (pH 7.5), and 62,5 µg/ml poly(dIdC)·poly(dIdC) (Sigma)) were diluted with distilled water in incubated for 10 minutes on ice. 20 fmol of Cy5 labeled probe was added, followed by another 20 minutes incubation on ice and afterwards separated by electrophoresis with a 5.2% nondenaturing polyacrylamide gel at 4°C and 250 V for 3 h in 0.5x TBE. For competition experiments, probe and competitor were premixed before addition of the protein mixture. For supershift experiments, antibodies were added 5 minutes after mixing probe and protein. After electrophoresis, probe was visualized using a Typhoon TRIO+ imaging station (GE Healthcare, Little Chalfont, United Kingdom). Antibodies used: SP1: Millipore (Billerica, Massachusetts, USA) 07-645; SP3: Santa Cruz (Santa Cruz, California, USA) sc-13918-X; PPARγ Millipore 07-466; RXRα: Santa Cruz sc-774-X.

### Western Blot

Total protein was extracted from cells and protein concentration was measured by the bichinconic acid method. 15 µg protein per lane was resolved on a 10% SDS-PAGE and transferred to a nitrocellulose membrane (Li-Cor, Lincoln, Nebraska, USA) using a semidry blotting apparatus (BioRad, Hercules, California, USA). Protein was subsequently targeted by SP1 and SP3 antibodies which were then detected using IR-Dye conjugated secondary antibodies. Images were acquired using a Li-Cor Odyssey imaging station and the manufacturer’s software.

### Statistical Analysis

Statistical analysis was performed using SigmaStat 3.5 (Systat Software, Chicago, Illinois, USA). Asterisks indicate a statistical difference. The exact test used is stated in the respective figure legend. Exact statistical p-values are given in the results text. Where necessary, data were log-transformed (log_10_ of Data+1). All p-values given in the text are unadjusted p-values but significant when adjusted for multiple testing.

### Image Processing

EMSA and Western Blot images were acquired by fluorescence scan using either a Typhoon Trio+ or Licor Odyssey operated by the manufacturers’ software, always ensuring that no part of the image was oversaturated. Image processing is limited to modification of brightness and contrast using either the respective devices’ software or ImageJ to obtain good visibility of all features of importance and was always carried out for the whole image. Bar Charts were generated using GraphPad Prism 4, line art was assembled in PowerPoint 2007.

## Results

### A Deletion Study Hints Towards a Complex Enhancer Region within the First Intron

Knowing about at least two regulatory elements located within the first intron of UCP3, we tested for further binding elements located nearby. We aligned the intronic sequences of mouse, Djungarian hamster (*Phodopus sungorus)* and rat to compare conservation around the intronic module to the remaining intron. The first intron of the hamster is shorter compared to mouse and rat corresponding to the first half of the introns in these species. Generally, sequence conservation was low across the first intron, except a region of high conservation ranging from IVS(intervening sequence)1+1200 to IVS1+1850 with the IVS1+1505G/A base exchange in the center of this region.

**Figure 1:**
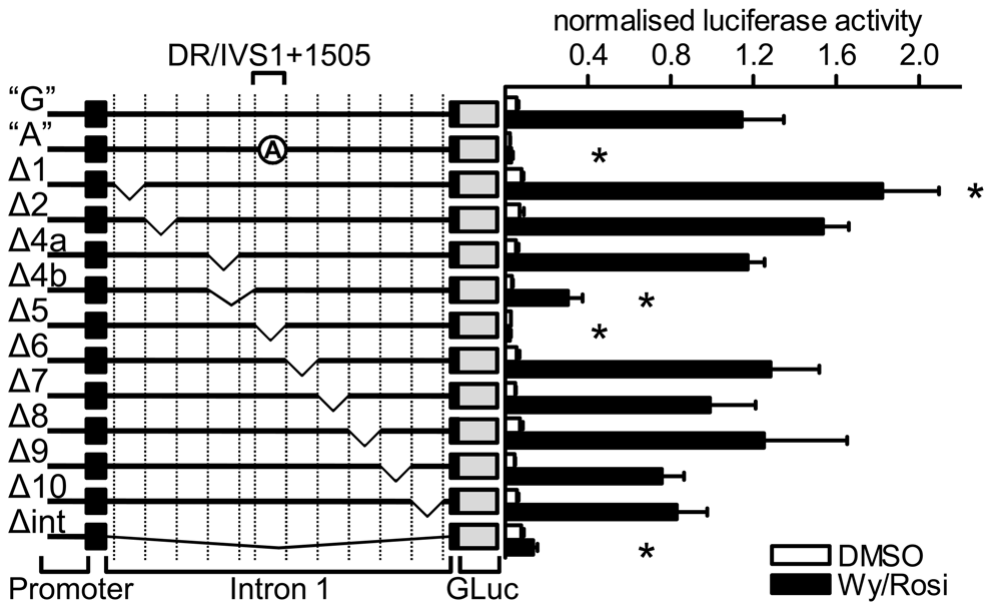
Stepwise deletion of the first intron reveals additional regulatory elements. Using PCR-mediated deletion, several 300–400 bp deletion covering most of the first intron in the IVS1+1505G (“G”) reporter gene construct were generated. All constructs were transfected into HIB1b brown adipocytes and exposed to a combination of Wy14643 (Wy, 10 µM) and rosiglitazone (rosi, 10 µM) or vehicle. Black boxes represent the first 2 exons of UCP3. Crossed circles represent mutation of the elements indicated above. GLuc: Gaussia Luciferase. n = 4 to 5 for Wy/Rosi and n = 3 for DMSO. Bars represent mean values ± s.d. Stars denote a significant difference from the IVS1+1505G vector in the presence or absence of agonists, respectively (two way ANOVA for construct and agonist, Holm-Sidak Method).

To discover regulatory elements in these conserved regions or beyond, we generated 11 constructs with sequential deletions of 300–400 bp within the first intron of the hamster reporter. All constructs as well as the IVS1+1505A (brown fat specific lack of UCP3 expression in hamster) and IVS1+1505G (wildtype hamster) constructs and a further construct lacking the entire first intron were transfected into the brown adipocyte cell line HIB1b. Twenty hours after transfection, the cells were exposed to either Wy14643 and rosiglitazone or vehicle (DMSO). The 300–400 bp deletion constructs were termed Δ1 to Δ10 (Figure 1). Construct Δ1 and Δ2 lack regions between the first exon and the conserved region described above. Δ4a and Δ4b are lacking the conserved region upstream of the DR/IVS1+1505 module and are mostly overlapping but differ in the proximity of the deletion to the DR1 element. Δ5 covers the module, while Δ6 covers the conserved region downstream of IVS1+1505. The deletions Δ7 to Δ10 cover the rest of the intron downstream of the conserved region to the second exon. The deletion ΔInt removes the first intron completely. Deletion of the Δ3 region was not successful.

We then compared all constructs to the IVS1+1505G and A constructs (“G” and “A”) (Figure 1). The reporter deleted for the DR1/IVS1+1505 module (Δ5) lost nearly all activity and did not respond to PPAR stimulation thus resembling the “A” construct. Deletion of the entire intron in construct Δint also caused strong repression, although not to the same extent as Δ5. All other constructs were PPAR agonist responsive and elicited significantly higher activity compared to the “A” construct. The constructs Δ2 and Δ7 to Δ10 were not different from the “G” construct, which is in line with the low conservation of the respective regions. Conversely, this was also true for Δ4a and Δ6 despite the high conservation of the region deleted. The only constructs whose agonist stimulated activity differed from the “G” construct and were responsive to PPAR agonists were Δ1 and Δ4b. For Δ1, the reporter activity upon stimulation is increased by 59% (p<.001) compared to “G”, implying a suppressor element within the first 400 bp of intron 1. Construct Δ4b activity was decreased by 74% (p<.001) indicating the binding of a transcriptional activator. In summary, a short intronic region including the previously described DR1 and IVS1+1505G elements specifically conferred transactivation of the UCP3 gene.

### *In Vitro* Binding of SP1 and SP3

To identify the transcription factors binding to the IVS1+1505G allele in vitro we employed EMSA. We validated specificity of the observed complexes by comparison of the IVS1+1505G and IVS1+1505A probes and by addition of unrelated non-labeled DNA competitors in molar excess. We defined complexes to be specific when they were both specific for the IVS1+1505G probe compared to the IVS1+1505A probe and did not diminish when competed by a NFκB (unrelated transcription factor) oligonucleotide.

In a previous study, we had already dismissed the family of forkhead transcription factors as candidates binding to IVS1+1505G [27]. We analyzed further transcription factors identified as candidates binding to this element by bioinformatic sequence analysis and designed competitor probes with the respective consensus binding motifs. However, none of the candidates tested was both detectable in our cell lines and proved to be able to compete complex formation on the IVS1+1505G probe (Figure S1). In addition, competitors resembling the hamster element but carrying mutations at different positions were assayed. We then compared the sequences of non-competing and competing oligonucleotides to pinpoint the crucial positions. These experiments revealed the GC rich streak within the IVS1+1505G probe that was indispensable for complex formation. Data mining on GC-boxes and expert advice (personal communication, Guntram Suske) hinted towards a SP1/3 binding motif [27].

**Figure 2:**
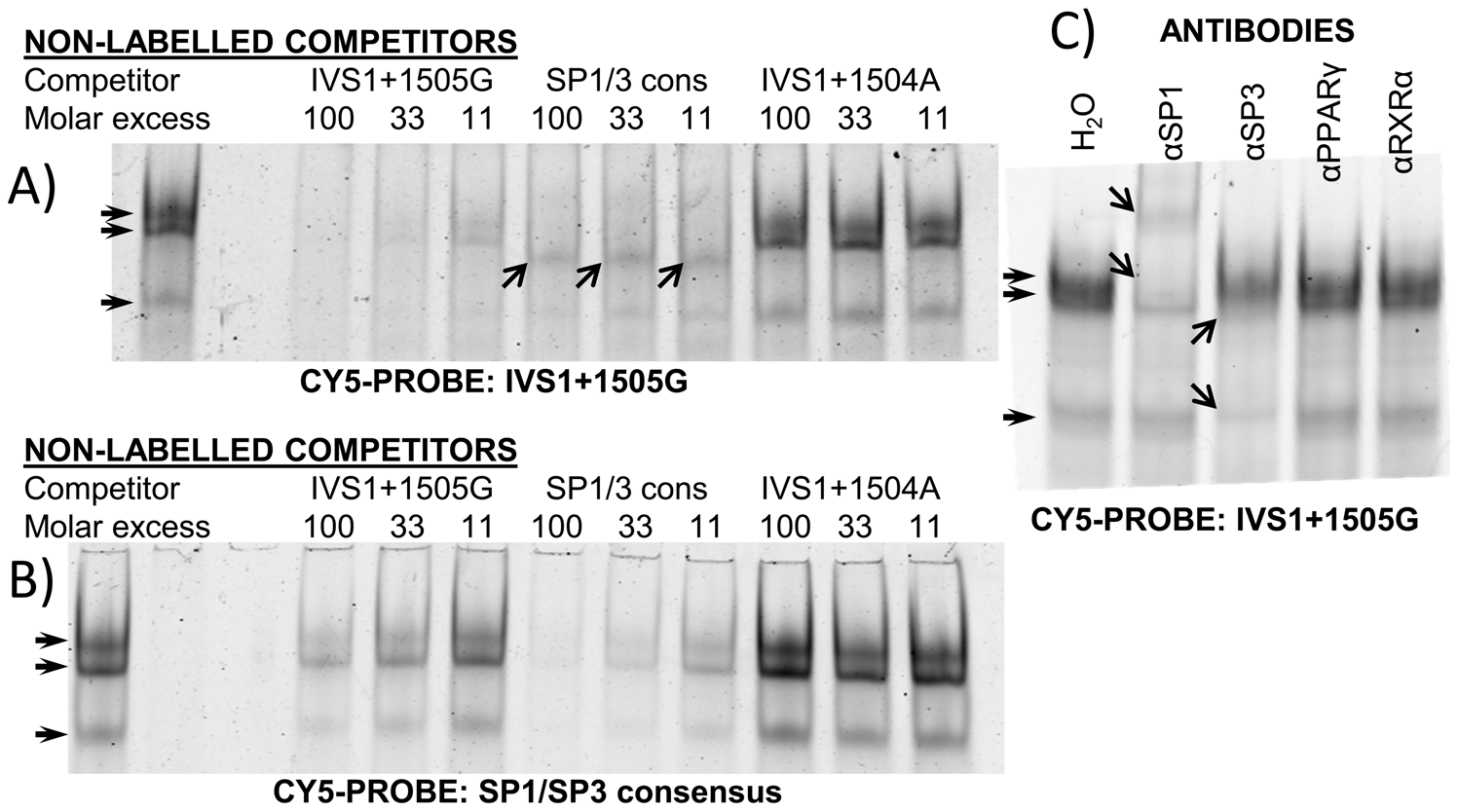
The IVS1+1505G element binds SP1 and SP3 in EMSA. EMSA bands were obtained incubating either the Cy5 labeled probes IVS1+1505G (A, lane 1) or SP1/SP3 consensus (B, lane 1) with nuclear extracts from HIB1b cells followed by native PAGE. Non-labeled competitors IVS1+1505G, IVS1+1504A and SP1/3 consensus were added to the binding reaction along with labeled probe where indicated. Different spacing between the complexes and a non-SP complex formed with the IVS1+1505G probe (arrows in (A), competition with SP1/3 consensus) hint to different complex compositions. (C) Supershift experiments by addition of antibodies against SP1, SP3, PPARγ and RXRα to test the identity of the proteins binding to the IVS1+1505G element. A representative experiment of 3 independent repetitions is shown in C.

To investigate a possible involvement of SP1 and SP3, both unlabeled and Cy5-labeled SP1/3-consensus probes as well as antibodies targeting SP1 and SP3 were applied. EMSA experiments using labeled IVS1+1505G probe with unlabeled SP1/3 consensus competitors and vice versa then demonstrated: Firstly, a competitor containing a SP1/3 binding GC-Box was able to impair complex formation on the IVS1+1505G probe when added in molar excess, most likely via binding and thereby depleting SP1 and SP3 from the binding mixture. Second, an unlabeled IVS1+1505G competitor impaired complex formation on a SP1/SP3 consensus probe, most likely via the same mechanism. This demonstrated that both probes essentially bound the same proteins (Figure 2A and B), except from an additional, yet unidentified complex formed with the IVS1+1505G probe after depletion of SP factors using a consensus competitor. Thirdly, addition of antibodies targeting SP1 and SP3 to the binding reaction shifted or disrupted complex formation, most likely via binding to proteins involved in the IVS1+1505G binding complex. This in vitro binding could be shown using several different antibodies/antisera targeting SP1 and SP3 (Figure 2C and Figure S5). Neither a PPARγ, nor a RXRα antibody influenced specific complex formation. Supershift experiments using a SP4 antibody and epitope-tagged versions of SP2 and CREB support specificity of SP1/3 binding (Figure S5 and S6). Taken together, these data demonstrate that SP1 and SP3 bind to the IVS1+1505 element in an allele specific manner in vitro.

### Binding of SP1 and SP3 to the IVS1+1505G Element is Essential for Expression of UCP3

We investigated the effect of RNAi mediated knockdown of SP1 and SP3 as well as the effect of a binding inhibitor in cell culture to verify that binding of SP1 and SP3 influences expression of UCP3.

We used virus-delivered miRNAs to deplete SP1 and SP3. Each virus delivered two different miRNA sequences. HIB1b cells were exposed to the retrovirus and subsequently selected by addition of puromycin to remove non-infected cells. We chose miRNAs targeting the LacZ and the shBle (Ctrl. Z) gene and two different miRNAs targeting UCP1 (Ctrl. U) as control conditions. For single SP1 or SP3 knockdown we combined two miRNAs targeting the respective gene, for the double knockdown we combined the most efficient SP1 miRNA with the most efficient SP3 miRNA. Knockdown was confirmed by western blotting (Figure S2). Knockdown of SP1 led to a compensatory increase of SP3 protein and vice versa.

**Figure 3:**
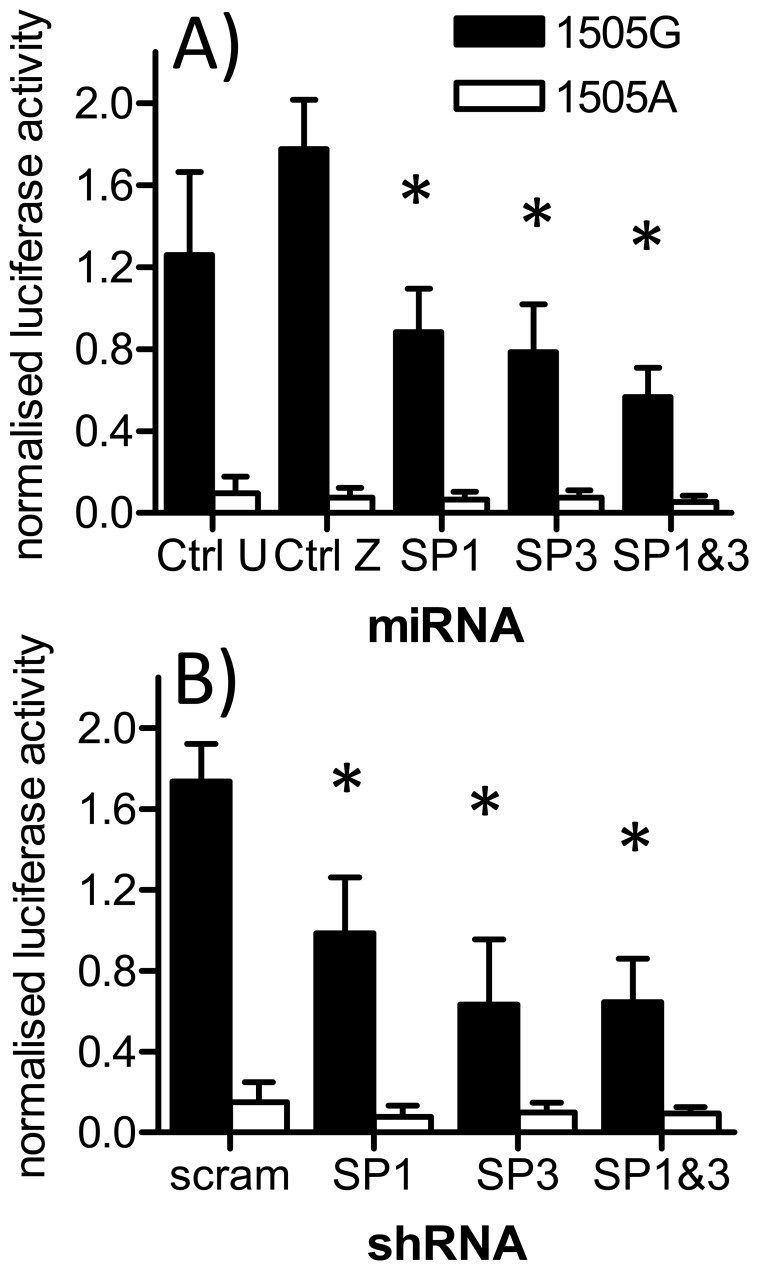
Targeting SP1 and/or SP3 via RNAi decreases reportergene activity of the IVS1+1505G reporter gene construct. miRNA-expressing HIB1b cells were transiently transfected with IVS1+1505G or A, induced and differentiated. During the last 24 hours of differentiation cells were stimulated by a combination of Wy14643 (Wy, 10 µM) and rosiglitazone (rosi, 10 µM). (A) Each cell line expresses two miRNAs targeting either twice SP1, twice SP3, each SP1&SP3 once, UCP1 (Ctrl. U, no transcript detectable in HIB1b cells) or LacZ/shBle (Ctrl. Z, two bacterial genes) The experiment was repeated 8 and 7 times for IVS1+1505G and IVS1+1505A, respectively, each time in triplicates using cells from 2 independent rounds of infection and selection. scram: scrambled shRNA sequence C) Replication of the miRNA experiment using transient transfection of shRNAs with independent sequences. The experiment was carried out 3 times in duplicates. Bars represent mean ± s.d. Stars denote a significant difference from both control vectors for the respective agonist (one way ANOVA for miRNA, Holm-Sidak method, Log transformed data).

Knockdown of either SP1 or SP3 led to 40% (SP1 vs Ctrl U: p<.01; SP1 vs. Ctrl Z: p<.001) and 47% (SP3 vs Ctrl U: p<.01; SP3 vs. Ctrl Z: p<.001 vs Ctrl U/Z) reduction in IVS1+1505G construct activity, respectively (Fig. 3A). Knockdown of both SP1 and SP3 reduced activity by 61% (SP1+SP3 vs Ctrl U: p<.001; SP1+SP3 vs Ctrl Z p<.001). All three knockdown conditions were significantly different from either control after adjusting for multiple testing. Conversely, even the double knockdown did not have a statistically significant effect on the mutant IVS1+1505A construct, and while there is a trend of towards a lower reporter activity, the effect size is low. For the single knockdowns of SP1 or SP3 reporter activity of the IVS1+1505A reporter was on the same level as the controls.

To validate the miRNA data and to exclude off-target effects we repeated the experiment with an alternative RNAi strategy (shRNAs). shRNA vectors were delivered by the Nucleofection transfection method. The results reproduce the effects described above and are shown in Figure 3C.

**Figure 4:**
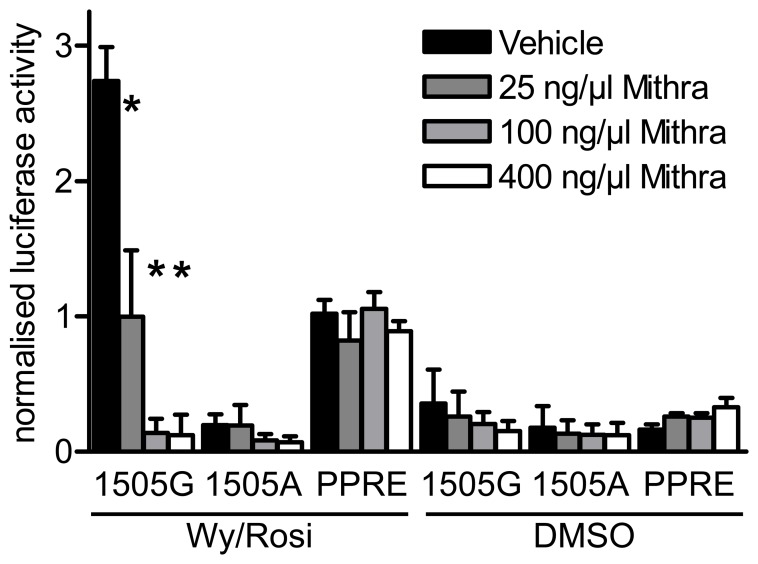
Mithramycin suppresses PPAR agonist mediated activation of the IVS1+1505G reporter gene construct. HIB1b cells were transiently transfected with the reporter gene vectors IVS1+1505G, IVS1+1505A or a 3xPPRE consensus element and subsequently stimulated by the PPAR agonists Wy14643 and Rosiglitazone (in combination, 10 µM each) or DMSO for 24 hours in presence or absence of different concentrations of Mithramycin. Mithramycin concentrations used were 25 ng/ml, 100 ng/ml and 400 ng/ml or no Mithramycin (DMSO/vehicle). Bars represent mean ± s.d. (one way ANOVA for Mithramycin concentration, Holm-Sidak method, Log transformed data).

We excluded that knockdown of SP1 and SP3 modulates IVS1+1505G reporter gene expression indirectly by adding the SP binding inhibitor mithramycin to cells either transfected with the IVS1+1505G construct, the IVS1+1505A construct, or a PPRE consensus reporter gene construct (Figure 4). Comparing the effect of mithramycin on reporter gene activity with or without stimulation by Wy14643 and rosiglitazone revealed that the stimulation by PPAR agonists can be impaired (25 ng/ml mithramycin, p<.001) or even abolished (100 ng/ml mithramycin: p<.001; 400 ng/ml: p<.001) by inhibition of SP binding to the IVS1+1505G construct. Mithramycin had no effect on the PPAR agonist mediated activation of the PPRE consensus construct, and no effect on IVS1+1505A, which was not inducible by PPAR agonists in the first place. The transcriptional activation by SP1/SP3 was thus dependent on the IVS1+1505G element located within the first intron while mithramycin does not interfere with signal transduction via PPREs in general. These effects seen in reporter gene assays are supported by the observation that mithramycin treatment also reduces expression of endogenous UCP3 in cell culture (Figure S7).

Taken together, the three sets of RNAi data and the mithramycin experiment provide very strong evidence that both SP1 and SP3 bind to the IVS1+1505G element and are indispensable for expression of UCP3 in brown fat cells. Additionally they suggest a functional interdependence between the SP binding element and the DR1 element within the first intron.

### The Intronic SP1/3 Element and the Intronic DR1 Element are Interdependent in their Function in Brown Adipose Tissue

To elucidate the contribution and the cooperativity of the promoter DR1 element, the intronic DR1 element and the intronic SP1/3 element we generated 8 vector constructs covering all possible combinations. We used quick change mutagenesis to delete either the promoter DR1, or the intronic DR1 or both for each the IVS1+1505G and IVS1+1505A constructs. These constructs were then transfected into HIB1b brown adipocytes which were subsequently treated with Wy14643 and rosiglitazone (stimulated) or DMSO (non-stimulated).

**Figure 5:**
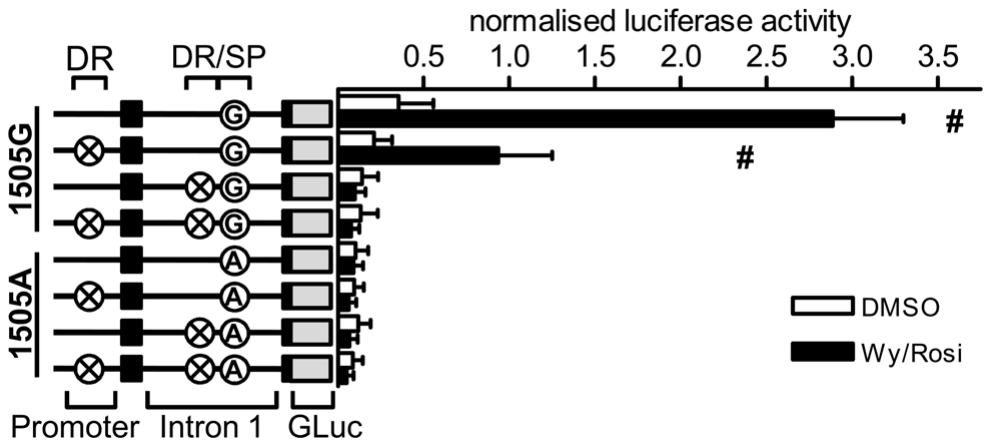
PPAR agonist-mediated UCP3 expression depends on combined presence of the intronic SPx/DR1 double element. In the IVS1+1505G and A reporter gene constructs either one or both of the two putative DR1 elements were mutated. The 8 constructs were transfected into HIB1b cells and exposed to Wy14643 and Rosiglitazone (in combination, 10 µM each) or DMSO in differentiation medium for 24 hours. Black boxes represent the first 2 exons of UCP3. Crossed circles represent mutation of the respective elements indicated above. Circles with “G” or “A” indicate the allele at the IVS1+1505 position in intron 1. GLuc: Gaussia Luciferase. N = 3–4 for Wy/Rosi and N = 2–3 for DMSO. Bars represent mean ± s.d. # marks constructs that respond to PPAR agonist stimulation compared to vehicle. (two way ANOVA for Vector and Agonist, Holm-Sidak Method).

Deletion of either DR1 element reduced reporter gene activity (Figure 5). The construct IVS1+1505G with both DR1 elements intact displayed the highest luciferase activity. Interestingly, the two DR1 elements contributed to a different extent to reporter gene expression. Deletion of the promoter DR1 in the IVS1+1505G construct resulted in a 68% reduction of stimulated activity, but the reporter activity remained responsive to PPAR agonists (p<.001). In contrast, mutation of either the intronic DR1 element or the intronic SP1/3 element (“A”) led to a complete loss in responsiveness to stimulation and in baseline reporter gene activity. This effect was independent of the presence of the promoter DR1 element. Repetition of the experiment in immortalized primary brown preadipocytes replicates these findings (Figure S3).

Strikingly, none of the two DR1 elements can confer PPAR ligand dependent activation without the presence of the intronic SP1/3 element (“G”). While the experiment cannot differentiate whether the SP1/3 element is indispensable for UCP3 expression per se, or only necessary for PPAR agonist activation of UCP3, both hypotheses underline the critical importance of the SP1/3 element.

### The SP/DR Module is a General Feature of the UCP3 Gene of Many Different Species

Using bioinformatic software (Genomatix Genome Analyzer), we screened the UCP3 genes of horse, rat and man for putative SP/DR modules. This approach identified one putative SP/DR module in the human gene, 2 putative elements for pig and 4 putative modules for horse. All putative modules were found in approximately the same distance from the respective transcriptional start site. Notably, the human element identified here is different from the one proposed by us previously [27] which is located in the first intron of UCP3. In the human gene, in which the first intron of UCP3 is shorter than in rodents, this distance places the module in exon 2 within coding sequence.

**Figure 6:**
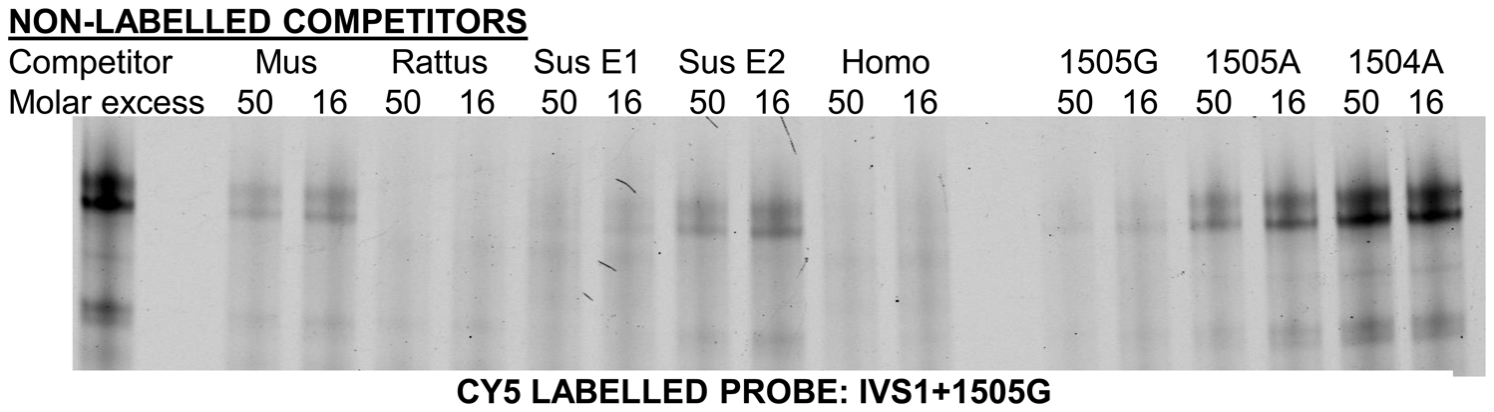
A downstream SP element is a common feature of many mammalian UCP3 genes. The UCP3 genes of mouse (*Mus*), rat (*Rattus*), pig (*Sus*) and human (*Homo*) were analyzed for SP/DR modules downstream of their promoter using the Genomatix software package. For all species one (mouse, rat, human) or two (pig, E1&E2) modules were predicted roughly 1500 bp downstream of the transcriptional start site. Oligonucleotides resembling the predicted SP site were annealed and used as cold competitor in EMSA against a Cy5 labeled *Phodopus* IVS1+1505G probe. As a negative control, IVS1+1504A, a probe lacking a crucial C of the GC-Box, does not compete at all. Shown is one representative EMSA out of at least 4 independent experiments.

To test the functionality of the putative modules, we used unlabeled competitors resembling the sequences of the putative SP1/3 element in EMSA experiments (Figure 6). For rat, mouse and human the predicted element was well able to compete with the hamster element. For pig, one of the elements was able to strongly compete, while the other element only had a mild effect on complex formation.

While we understand that simple EMSA experiments are not sufficient to validate presence of a complex transcription factor binding module conserved across the whole mammalian class, our data provide good evidence that it an intronic enhancer in the first intron of the UCP3 gene is of importance in non-rodent species as well. The relevance of downstream elements in the regulation of the human UCP3 gene is supported by a deletion study, although the authors were unable to pinpoint distinct elements or mechanisms [32]. To explore possible disease relevance, the putative DR/SP element was sequenced in in 95 obese children and adolescents and 96 underweight adult subjects. While we could not identify any group differences, we found the region to show low variation, hinting towards functional conservation. Details on the analysis and the underlying cohort [33] can be found in Method S1.

### ChIP-seq Data Reveal the Involvement of MyoD, Myogenin and p300 at the Intronic Enhancer Module

**Figure 7:**
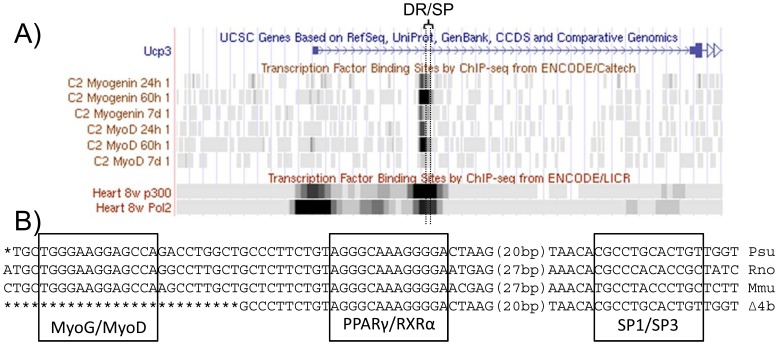
ChIP-seq Data from C2C12 cells demonstrate MyoD binding to the region deleted in Δ4b. (A) Publicly available ChIP-seq data for MyoD and Myogenin in C2C12 cells were mapped to the first intron of UCP3. Interestingly, only one of these experiments demonstrates MyoD binding to the promoter of UCP3. Furthermore, ChIP-seq data for the co-activator p300 and RNA polymerase 2 were mapped. Screenshot taken from the ENCODE browser. ChIP-seq data for BAT was not available. (B) Alignment of the three intronic binding elements in hamster (Psu), rat (Rno) and mouse (Mmu). Intronic sequences were obtained from ENSEMBL (www.ensembl.org). Putative binding elements are marked by boxes. The fourth row of sequence resembles the *Phodopus* reporter gene construct carrying the deletion Δ4b. Shown are 25 bp of 36 bp deleted in Δ4b, but not in Δ4a. Numbers in brackets denote bases left out for the sake of clarity.

Due to the fact that Δ4a and Δ4b only differ by 36 bp deleted in Δ4b, but not in Δ4a, the region attributable for the difference in reporter gene activity is very small (Figure 1). Using publicly available ChIP-seq data supplied by the ENCODE project we analyzed the region for binding sites (Figure 7A). As there were no data available on brown adipocytes, we inspected data from heart and C2C12 myotubes. These data revealed binding of both MyoD and Myogenin, which are both preferentially expressed in muscle but only weakly expressed in BAT, and the coactivator p300, which is widely expressed, within 100 bp upstream of the DR/SP module within intron 1. Interestingly, these factors were all published to act via the core promoter of UCP3, but according to ChIP-seq data preferentially bind to the intronic region identified here and not to the promoter. Polymerase II, in contrast, is mainly found on the promoter of UCP3.

Comparing the ChIP-seq data to the sequences deleted in Δ4a and Δ4b, the region the two constructs differ co-locates with the ChIP-seq peak. Our subsequent sequence analysis revealed a consensus NF1/Myogenin binding site located within the 36 bp sequence deleted on the construct with diminished activity, Δ4b, but was present in the construct Δ4a, which showed WT-like reporter activity (Figure 7B).

### Comparison of RNA-Seq Data on Tissue Distribution of PPAR Expression

The IVS1+1505G→A mutation in hamster leads to loss of expression in BAT only while SKM expression seems to be nearly unaffected. The simplest explanation is binding of a BAT specific transcription factor that is absent in SKM. To identify candidate proteins in an unbiased approach we searched for such transcription factors that are expressed in BAT, but not in SKM, in publicly available datasets of expression profiling by high throughput sequencing (Gene expression omnibus, GEO). We chose sample GSM789832 of datasets GSE31843 (gastrocnemius muscle) and sample GSM929703 of dataset GSE36026 (brown adipose tissue). Original data files were mapped and compared by the Genomatix Mining Station and Genomatix Genome Analyzer software, respectively (Genomatix). Transcription factor transcripts (GO term „regulation of sequence-specific DNA binding transcription factor activity”) significantly overrepresented in BAT as compared to SKM included Cebpa (111-fold), Pparg (111), SOX5 (18,4), Gata6 (27,9), Irf4 (10,6), Ppara (7,5) and Hif1a (5,7). Of these, Cebpa and Pparg were by several orders of magnitude more abundant in BAT than all other transcripts. There was no significant difference for Ppard.

## Discussion

We previously identified a *cis* regulatory element located in the first intron of the uncoupling protein 3 (UCP3) gene of the Djungarian hamster [27]. A comparable element is also present in mouse, rat and human. In this element a naturally occurring sequence variation, intervening sequence 1 (IVS1) +1505G→A, completely disrupts UCP3 gene expression in brown adipose tissue (BAT) of the hamster, but only mildly impairs expression in skeletal muscle (SKTM). Comparing primary brown adipocyte cultures established from wildtype and mutant hamsters the peroxisome proliferator activated receptor (PPAR) agonist mediated stimulation of UCP3 gene expression is diminished in the mutant [23]. In reporter gene assays we confirmed that IVS1+1505G is essential for the action of PPAR agonists on UCP3 transactivation. We therefore aimed to identify the transcription factors which bind to IVS1+1505G and convey PPAR mediated regulation of UCP3 gene expression.

We discovered that the transcription factors SP1 and SP3 were binding to the IVS1+1505G element, whereas binding to the mutant allele was strongly diminished. Direct binding of PPARγ and RXRα to the IVS1+1505G element could be ruled out. Knockdown as well as chemical inhibition (mithramycin) of SP1 and SP3 in brown adipocytes impaired PPARγ agonist mediated transactivation of UCP3. Deletion of the region containing the putative SP factor binding element flanking IVS1+1505G supported the hypothesis that it is essential for the action of PPARγ agonists on UCP3 transcription and contains activator binding sites.

This interaction was surprising because the DR1 element conveying PPAR activation had previously been annotated in the core promoter, roughly 1600 bp upstream of IVS1+1505G, and this element is mainly sensitive to PPARα and PPARδ agonists [21]. Notably, a ChIP-seq screen for PPARγ binding in murine 3T3-L1 adipocytes localized a novel intronic DR1 element 40 bp upstream of IVS1+1505G [29]. Sequence alignment of rat, mouse and hamster uncovered conservation of both elements.

In our present study selective deletion of this DR1 and the SP element in reporter gene constructs revealed a functional interdependence between SP1/3 binding and PPAR agonist action. In brown adipocytes PPAR stimulation depended on the presence of both intronic DR1 and SP elements. Deletion of either element had far greater impact on PPARγ responsiveness of UCP3 in brown adipocytes than deletion of the promoter DR element. This indicates that the first intron of the UCP3 gene contains a SP/DR module conveying transactivation by PPARγ and the activity of PPARγ strictly depends on binding to the IVS1+1505G element. This finding is supported by the fact that SP1 and PPARγ have been reported to directly interact [34]. As of yet we can only speculate about the molecular mechanics behind this interdependence, but we consider 3 main hypotheses: Firstly, PPAR and RXR may not be able to bind their intronic element by themselves, but rather depend on other factors that prime/stabilize DNA binding. These factors would be SP1/SP3 in BAT and MyoD/Myogenin in skeletal muscle. This hypothesis would explain the tissue specificity of the IVS1+1505 polymorphism in *Phodopus*. Secondly SP1 and SP3 might facilitate DNA bending and thus bring the intronic enhancer into contact with the core promoter. PPAR and RXR could bind their binding site even in absence of SP transcription factors, but would not come into contact with the core promoter. A third hypothesis is that SP1 and SP3 are required for opening the chromatin, most likely via recruitment of p300, possibly in concert with PPAR and RXR.

Comparative genomics revealed that SP/DR modules in the UCP3 gene are conserved across several mammalian species. In the human UCP3 gene we found such a module within the second exon. Additionally, we found SP/DR modules within intron 1 of pig (*Sus scrofa domestica*) and horse (*Equus caballus*). All these modules are located in comparable distance downstream of the transcriptional start site. For rat, mouse, human and pig, we demonstrated the putative SP element of these modules to bind SP1 and SP3 using EMSA.

The essential role of the intronic SP/DR module for PPAR transactivation of UCP3 demonstrated in the present study is conflicting with previous findings suggesting PPAR action through a DR1 element in the promoter, located 50 bp upstream of the transcriptional start site [21]. This promoter DR1 element has been implicated to confer PPARα and δ agonist activity in BAT. Data from animal studies [22] and experiments in cell culture had repeatedly demonstrated PPARγ transactivation of UCP3 transcript [26]. Reporter gene experiments using the UCP3 promoter indicated involvement of PPARα and PPARδ, but could not reproduce the PPARγ effect [21]. Retrospectively, absence of the first intron in these reporter gene constructs probably explains the difference. Using our reporter constructs including the first intron, we assayed the involvement of different PPAR factors using specific agonists for PPARα (Wy14643), PPARγ (rosiglitazone) and PPARδ (GW0742). Rosiglitazone led to near maximal induction of UCP3 reporter gene activity at concentrations as low as 80 nM (Figure S4), while Wy14643 and GW0742 only were effects at concentrations more than a 1000-fold of their respective EC50 values. We hypothesize that UCP3 in BAT is mainly regulated by PPARγ via the intronic element and by PPARα via the core promoter. This is well in line with the literature: On the one hand experiments focusing on endogenous transcript in both rat and cell culture demonstrate PPARγ agonist induction of UCP3 transcription [26], [35]; on the other hand experiments employing reporter constructs lacking the first intron find only PPARα but not PPARγ agonist effects [21]. Notably, this divergence between reporter gene data and endogenous transcript data could not be explained without knowledge about the intronic regulatory elements. We are aware that there is still divergence between our reporter data and the literature regarding the lack of PPARα agonist effects in Figure S4, hinting that the effect of the combined rosiglitazone/Wy14643 treatment is entirely caused by PPARγ activity. Whether this is due to the lack of the respective elements in the reporter gene constructs or due to the cell culture system we cannot decide based on our data.

We expanded our deletion experiments to systematically search for further intronic transcription factor binding elements. While most of these constructs elicited similar reporter activity, one revealed a putative activator binding element 30 to 50 bp upstream of the intronic DR1 element. Publicly available ChIP-seq data demonstrate the binding of MyoD and Myogenin to this third important element in C2C12 cells. Further *in silico* analysis revealed the binding of the coactivator p300 to this element in heart and C2C12 cells, thus providing a possible mechanism to achieve tissue specificity. Notably recruitment of p300 by SP containing complexes has been demonstrated [36]. Comparing the ChIP-seq peaks of MyoD, Myogenin and p300 between promoter and intron, all three factors display stronger signals in the intron. Interestingly, this is well in line with the finding that all three deletions within the intronic enhancer region (the mutation in the SP1/3 element, the ablation of the intronic DR1 element, the deletion of the intronic MyoD/Myogenin element) led to a more pronounced reduction in reporter gene activity than the deletion of the promoter DR1 element. Correspondingly, the UCP3 reporter construct deleted for the entire intron showed both low activity and PPAR responsiveness in HIB1b cells. Solanes *et al.* [21] reported that constructs only harboring the promoter of UCP3 require overexpression of several transcription factors to become active and PPAR responsive, while our construct, covering both promoter and intron was responsive without the need for any overexpression and yielded a stronger fold induction upon agonist treatment.

**Figure 8:**
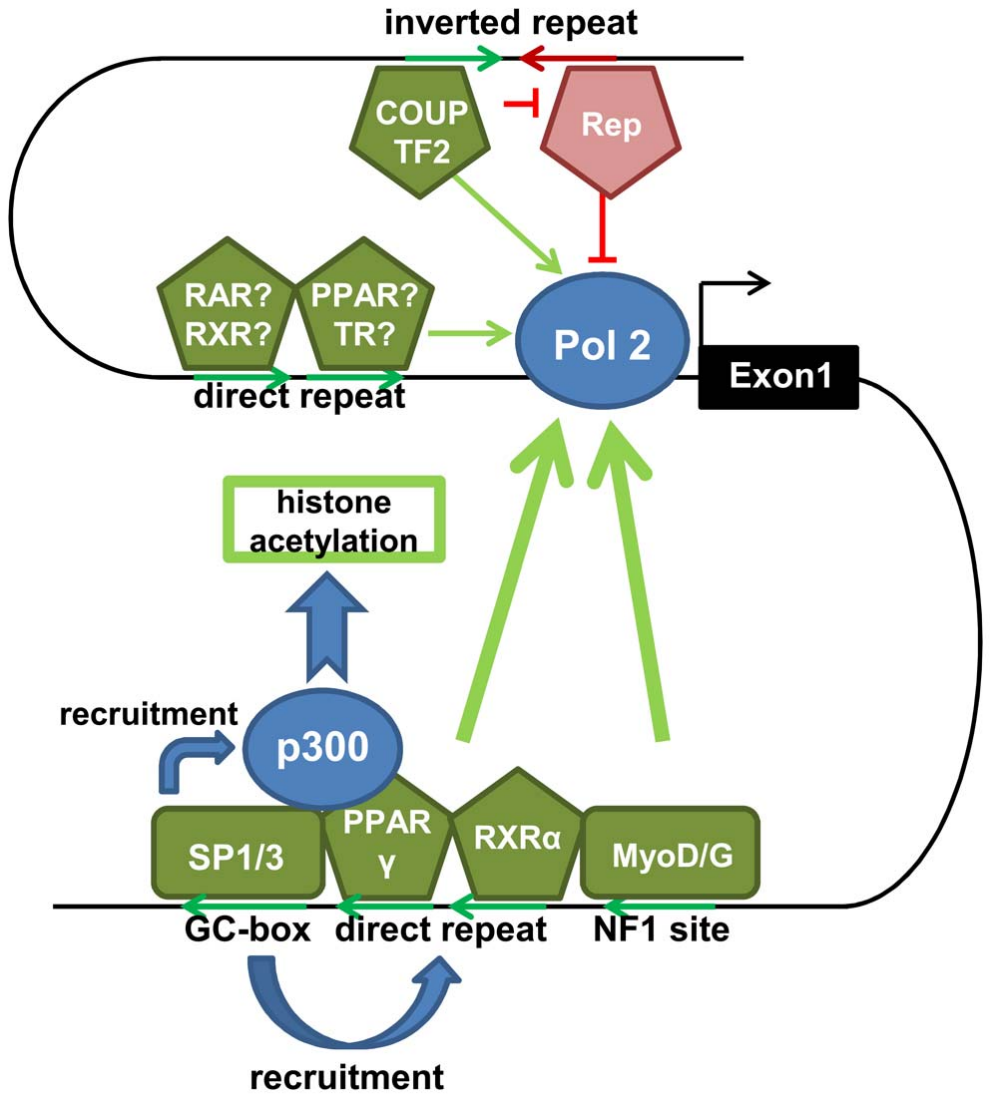
Regulation of UCP3 expression: Refined model. SP1 and SP3 bind to the intronic GC-box and recruit, in presence of the respective agonists, PPARγ and RXRα to the intronic DR1 element. This complex then recruits p300 to open the chromatin and enables initiation of transcription. Factors binding to the nearby NF1 site, (at least in muscle: MyoD and MyoG) join the complex and further increase the activating potency. The three intronic elements then, in cooperation with promoter elements and an upstream regulatory inverted repeat, regulate the expression of UCP3.

In conclusion, (see Figure 8 for schematic diagram including upstream enhancer sequences [37]) while our initial hypothesis, the presence of a single BAT specific transcription factor binding site, had to be discarded, we uncovered an intronic enhancer region located 1500 bp downstream of the transcriptional start site of the UCP3 gene. This enhancer is conserved across several mammalian species and depends on the activity of an SP-binding GC-Box/DR1 double element. Both elements are completely interdependent and indispensable for UCP3 expression and cannot perform their function without each other. The enhancer requires binding of SP1, SP3, PPARγ and RXRα, at least in BAT. For PPARγ, which has been previously published to bind to the promoter, our data demonstrate that in fact the intron is the main site of action. Interestingly the SP transcription factors seem to function as a gatekeeper, possibly via recruiting other components of the complex or mediating interaction with the core promoter and the transcriptional start site. Employing a deletion screen we pinpointed a MyoD/NF1 site located directly adjacent. The intronic UCP3 enhancer also recruits p300, thereby increasing chromatin acetylation. Previous publications proposed most of these interactions to take place at the core promoter, but our experiments and publicly available ChIP-seq data suggest that this has to be dismissed. Based on this new knowledge the current view on the regulation of UCP3 expression must be revamped: The first intron harbors a complex enhancer region, the UCP3 enhancer, and this enhancer is the dominant site for transcriptional regulation of UCP3 expression.

## Supporting Information

Figure S1 Consensus element competition scree for factors binding the IVS1+1505G probe. Unlabeled doublestranded oligonucleotides were tested in EMSA for their ability to bind the proteins that are forming the IVS1+1505G-specific complex and thereby to diminish complex formation. Of all tested consensus binding sequences only the CdxA consensus influenced the complex. Kons31 denotes the 31 bp consensus sequence generated by alignment of the first introns of UCP3 from several mammalian species that was carried out before identification of the GC-box.
(TIF)

Figure S2 Virus-delivered miRNAs decrease SP1 and SP3 protein amount in HIB1b cells. HIB1b cells were infected with different retroviral supernatants (indicated above). After puromycin selection cells were differentiated for 4 days and total protein was extracted. 20 µg per lane were separated on SDS PAGE and western blot was carried out with antibodies against SP1, SP3 and pan-actin (indicated left). NI: non infected/no selection/no miRNAs; ctrl Z: virus expressing 2 control miRNAs; SP1∶2 miRNAs targeting SP1; SP3∶2 miRNAs targeting SP3; SP1&3a one miRNA targeting each SP1 and SP3; SP1&3b: same as SP1&3a, but different miRNAs, ctrl U: 2 miRNAs targeting UCP1; GFP: overexpression of GFP, no miRNAs. Underlined miRNA cell lines were used for reportergene assays. Shown is a representative experiment of more than 4 independent blots.
(TIF)

Figure S3 Interdependence of SP1/3 binding and PPARγ agonist activity in SV40-LTA immortalized primary brown preadipocytes. Five of the reporter constructs used for the experiments shown in Figure 5 were transfected into immortalized preadipocytes and stimulated for 24 h with Wy14643 and Rosiglitazone. Immortalized cells were kindly provided by Patrick Seale. N = 3.
(TIF)

Figure S4 Only PPARγ ligands activate the IVS1+1505G reporter in a specific manner. The IVS1+1505G reporter gene construct was transfected into HIB1b cells and exposed to different agonist concentrations or DMSO for 24 hours in differentiation medium. Rosiglitazone, GW0742 and Wy14643 were added in the stated concentrations. According to the manufacturer (Cayman Chemical) the agonist concentrations required for receptor activation are 100 nM Wy14643 for PPARα, 30/100 nM Rosiglitazone for PPARγ1/2 and 1,1 nM GW0742 for PPARδ. The experiment was carried out once in triplicate wells.
(TIF)

Figure S5 Different, independent antibodies shift the SP1 and SP3 complexes in EMSA. HIB1b nuclear extracts were incubated with IVS1+1505G probe and different antibodies targeting either SP1 (Lane2: rabbit SP1 immune serum; Lane3: Millipore ABE135; Lane4: Santa Cruz sc-14027x) or SP3 (Lane5: rabbit SP3 immune serum; Lane 6: Santa Cruz sc-13018x; Lane7: Santa Cruz sc-644x). Lane1 contains no antibody, Lane8 contains SP4 Antibody (Santa Cruz sc-645x) and Lane 9 contains rabbit preimmune serum. Red arrows denote supershifts while blue arrows denote depleted complexes.
(TIF)

Figure S6 Epitope tagged versions of SP1 and SP3, but not CREB and SP2 bind to the IVS1+1505G probe. Immortalised brown adipocytes were infected with retrovirus expressing the full lenght cDNA of either CREB, SP1, SP2 or SP3 that were fused to a 2x Ty1 Tag at their N-terminus. Cells were used to generate RIPA extracts for a Western Blot (A) and nuclear extracts to perform EMSA supershift experiments (B). Red arrows denote the specific signals/supershift.
(TIF)

Figure S7 Mithramycin treatment decreases abundance of UCP3 Protein in immortalised brown adipocytes. Immortalised brown preadipocytes were induced and differentiated until full differentiation and and treated with Wy14643 (5 µM, PPARα agonist), Rosiglitazone (5 µM, PPARγ agonist), GW0742 (0,4 µM, PPARδ agonist) and All-Trans-Retinoic Acid (5 µM, RXR/RAR agonist) in presence or absence of 0,4 µM Mithramycin for 30 hours. RIPA extracts were generated and a Western blot against UCP3 (Pierce PA1-055), panAktin and CoxIV was performed. 40 µg protein were loaded per lane. Dottet lines indicate that the membrane was cut into 3 pieces.
(TIF)

Table S1 Oligonucleotides used for deletions/mutagenesis.
(DOC)

Table S2 miRNA sequences, top strand of 2 complementary oligonucleotides.
(DOC)

Table S3 miRNA combinations in the different viral constructs.
(DOC)

Table S4 Primers for amplification of the GFP+miR cassette for transfer into pMXs.
(DOC)

Table S5 shRNA sequences in pTER.
(DOC)

Table S6 Top strands of probes and competitors used in EMSA.
(DOC)

Table S7 Sequencing primers used for validation of constructs.
(DOC)

Table S8 Oligonucleotides for construction of tagged overexpression Vectors.
(DOC)

Method S1 Human sequence variations.
(DOCX)
