# Supplementary figures and images for: A Novel SP1/SP3 Dependent Intronic Enhancer Governing Transcription of the UCP3 Gene in Brown Adipocytes
Source: PLoS One. 2013 Dec 31;8(12):e83426. doi: 10.1371/journal.pone.0083426 (PMC3877035; doi:10.1371/journal.pone.0083426)

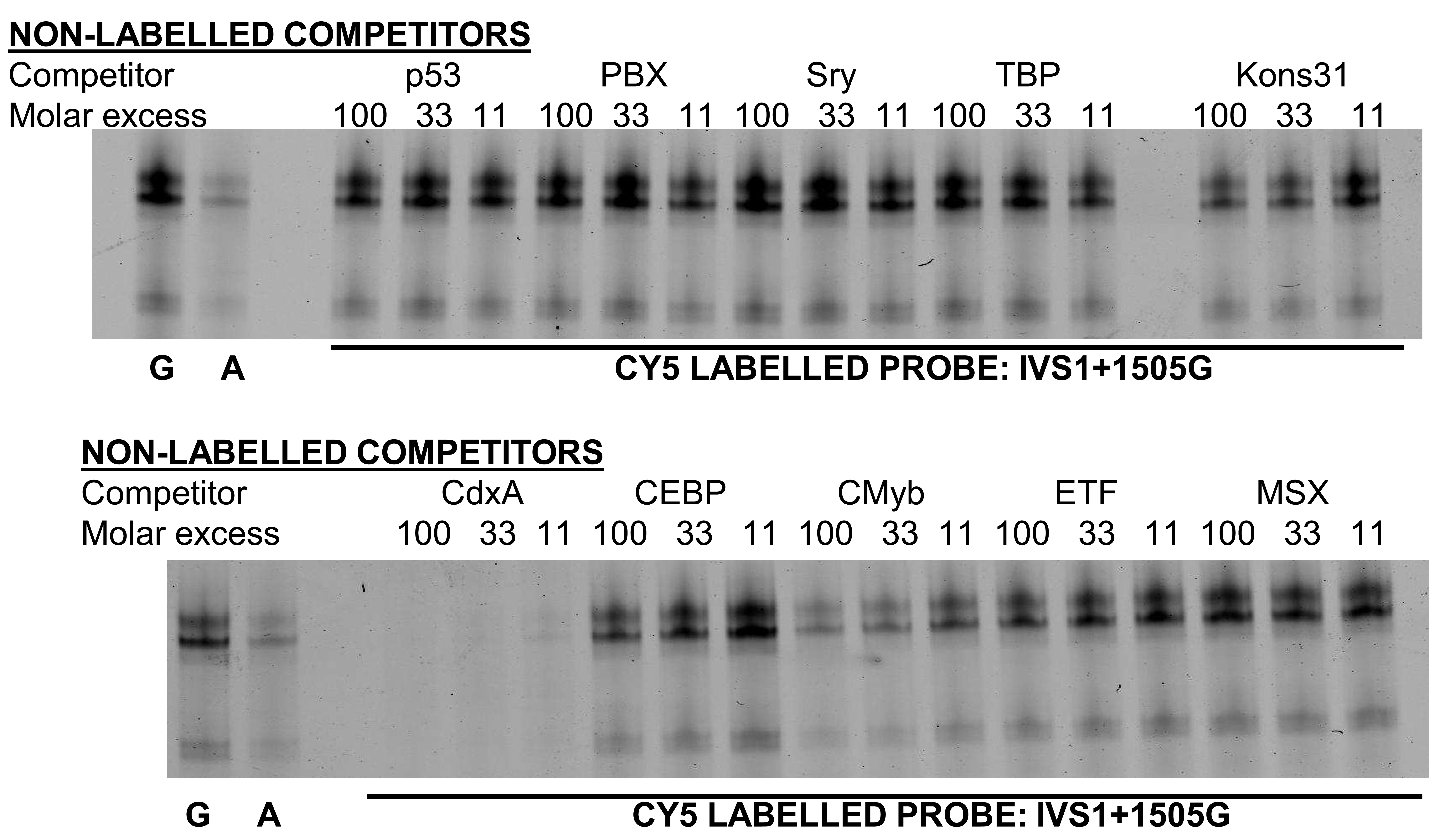

Supplement: Figure S1 — Consensus element competition scree for factors binding the IVS1+1505G probe. Unlabeled doublestranded oligonucleotides were tested in EMSA for their ability to bind the proteins that are forming the IVS1+1505G-specific complex and thereby to diminish complex formation. Of all tested consensus binding sequences only the CdxA consensus influenced the complex. Kons31 denotes the 31 bp consensus sequence generated by alignment of the first introns of UCP3 from several mammalian species that was carried out before identification of the GC-box. (TIF) [file pone.0083426.s001.tif]

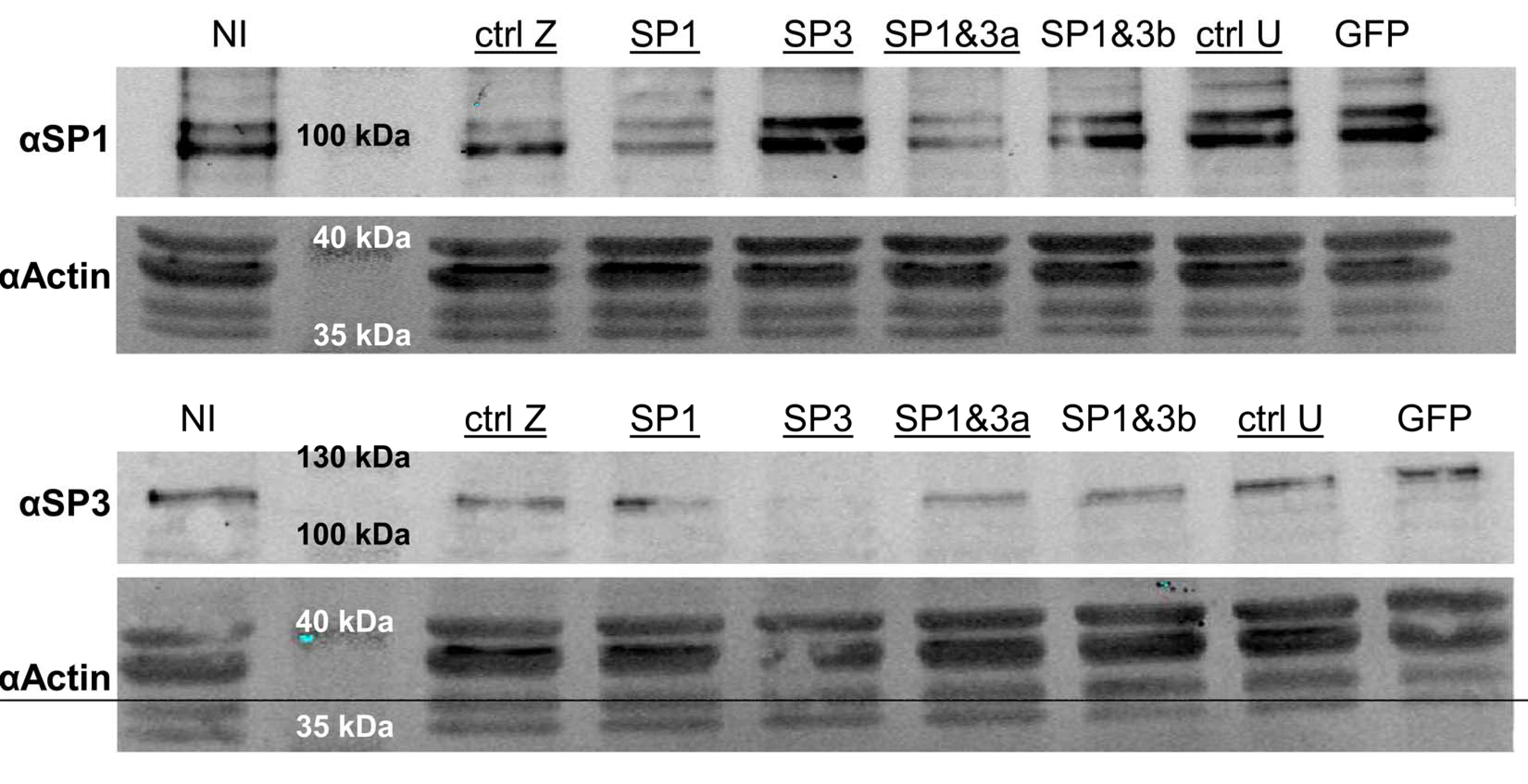

Supplement: Figure S2 — Virus-delivered miRNAs decrease SP1 and SP3 protein amount in HIB1b cells. HIB1b cells were infected with different retroviral supernatants (indicated above). After puromycin selection cells were differentiated for 4 days and total protein was extracted. 20 µg per lane were separated on SDS PAGE and western blot was carried out with antibodies against SP1, SP3 and pan-actin (indicated left). NI: non infected/no selection/no miRNAs; ctrl Z: virus expressing 2 control miRNAs; SP1∶2 miRNAs targeting SP1; SP3∶2 miRNAs targeting SP3; SP1&3a one miRNA targeting each SP1 and SP3; SP1&3b: same as SP1&3a, but different miRNAs, ctrl U: 2 miRNAs targeting UCP1; GFP: overexpression of GFP, no miRNAs. Underlined miRNA cell lines were used for reportergene assays. Shown is a representative experiment of more than 4 independent blots. (TIF) [file pone.0083426.s002.tif]

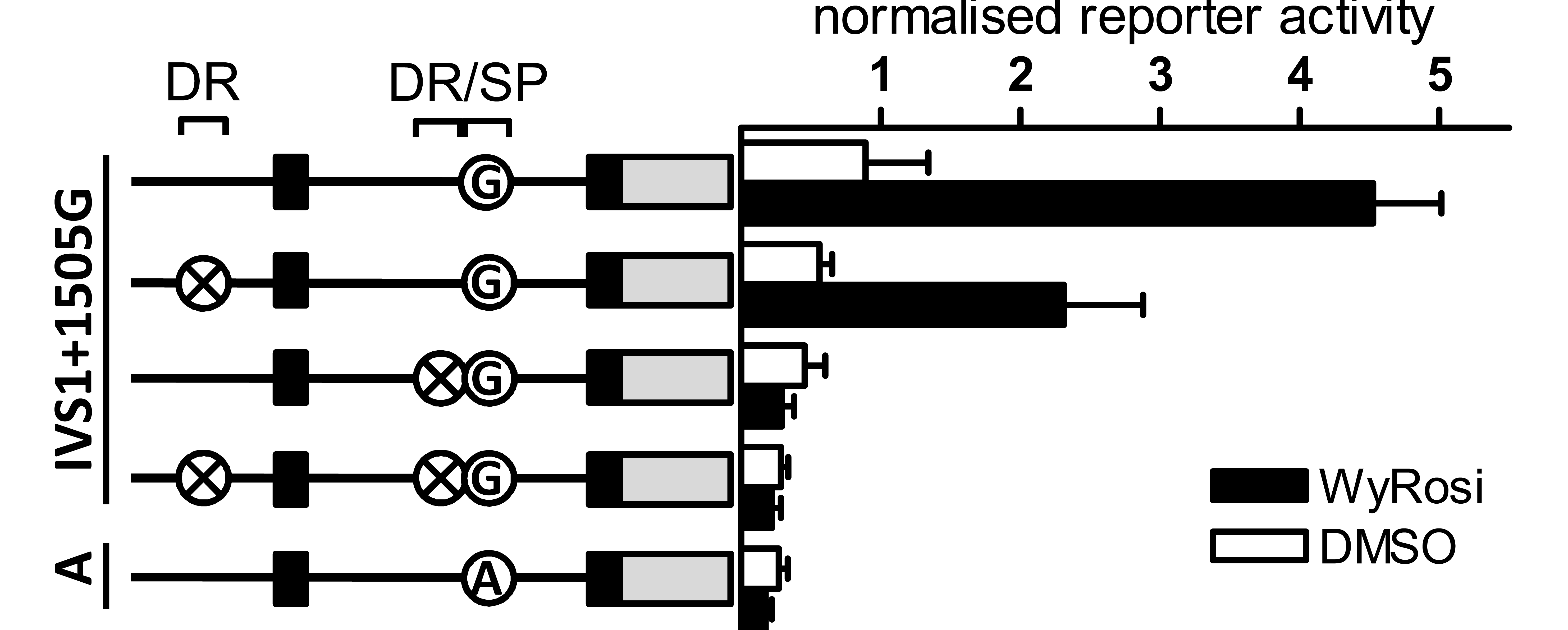

Supplement: Figure S3 — Interdependence of SP1/3 binding and PPARγ agonist activity in SV40-LTA immortalized primary brown preadipocytes. Five of the reporter constructs used for the experiments shown in Figure 5 were transfected into immortalized preadipocytes and stimulated for 24 h with Wy14643 and Rosiglitazone. Immortalized cells were kindly provided by Patrick Seale. N = 3. (TIF) [file pone.0083426.s003.tif]

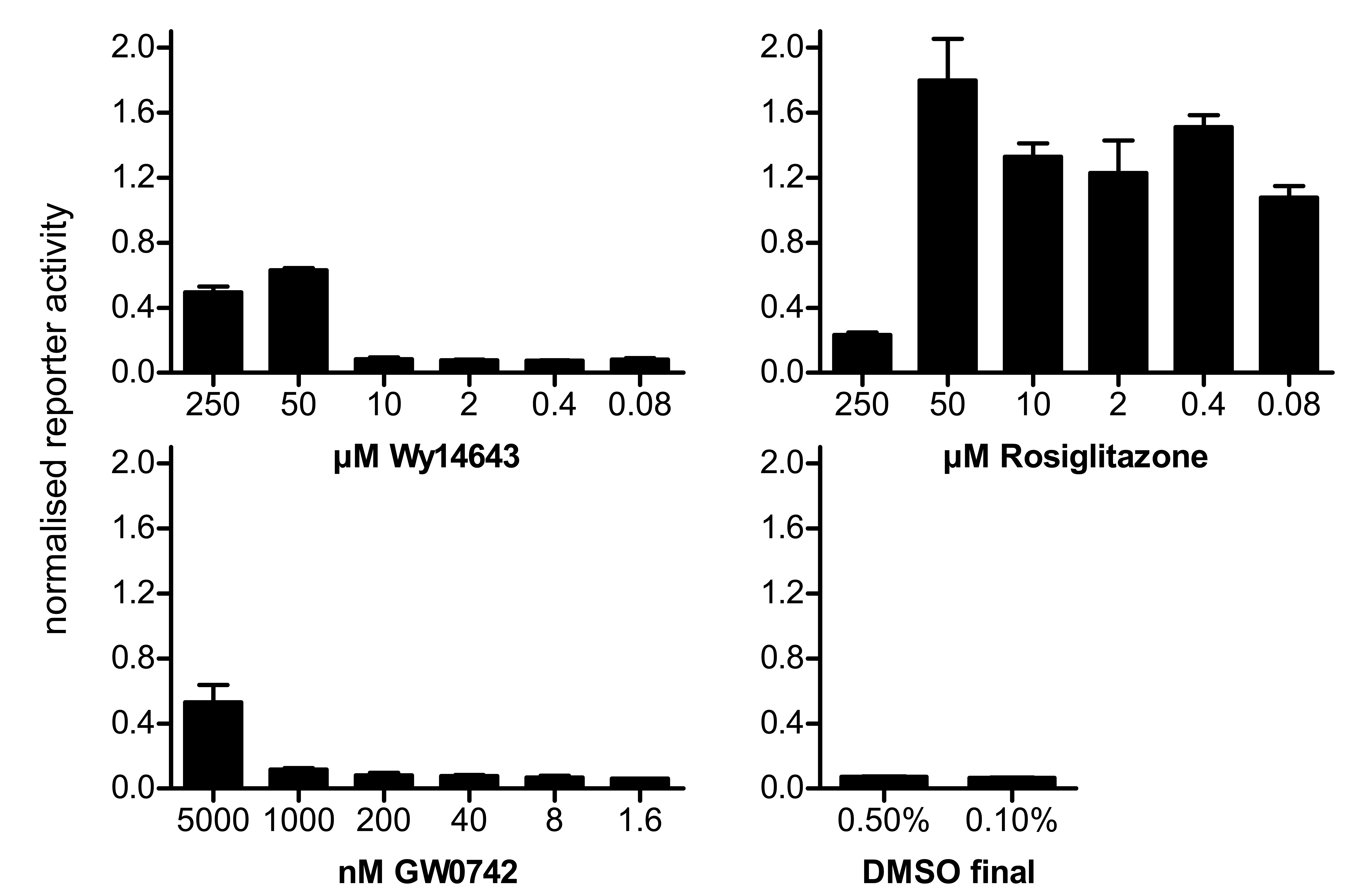

Supplement: Figure S4 — Only PPARγ ligands activate the IVS1+1505G reporter in a specific manner. The IVS1+1505G reporter gene construct was transfected into HIB1b cells and exposed to different agonist concentrations or DMSO for 24 hours in differentiation medium. Rosiglitazone, GW0742 and Wy14643 were added in the stated concentrations. According to the manufacturer (Cayman Chemical) the agonist concentrations required for receptor activation are 100 nM Wy14643 for PPARα, 30/100 nM Rosiglitazone for PPARγ1/2 and 1,1 nM GW0742 for PPARδ. The experiment was carried out once in triplicate wells. (TIF) [file pone.0083426.s004.tif]

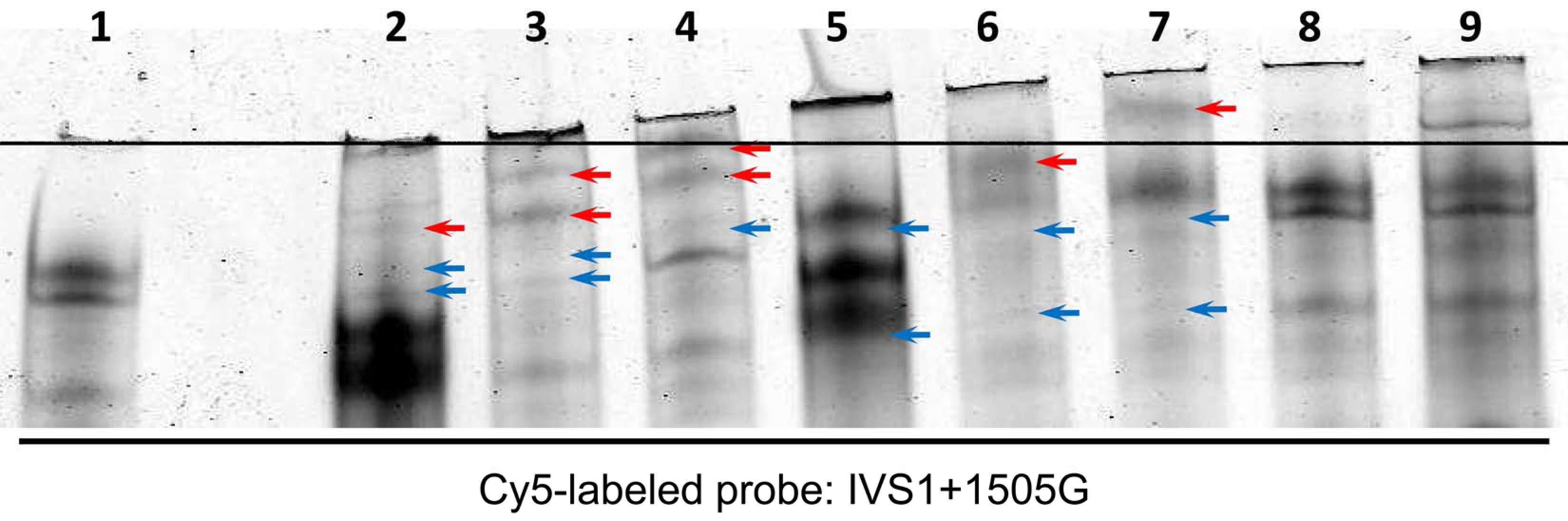

Supplement: Figure S5 — Different, independent antibodies shift the SP1 and SP3 complexes in EMSA. HIB1b nuclear extracts were incubated with IVS1+1505G probe and different antibodies targeting either SP1 (Lane2: rabbit SP1 immune serum; Lane3: Millipore ABE135; Lane4: Santa Cruz sc-14027x) or SP3 (Lane5: rabbit SP3 immune serum; Lane 6: Santa Cruz sc-13018x; Lane7: Santa Cruz sc-644x). Lane1 contains no antibody, Lane8 contains SP4 Antibody (Santa Cruz sc-645x) and Lane 9 contains rabbit preimmune serum. Red arrows denote supershifts while blue arrows denote depleted complexes. (TIF) [file pone.0083426.s005.tif]

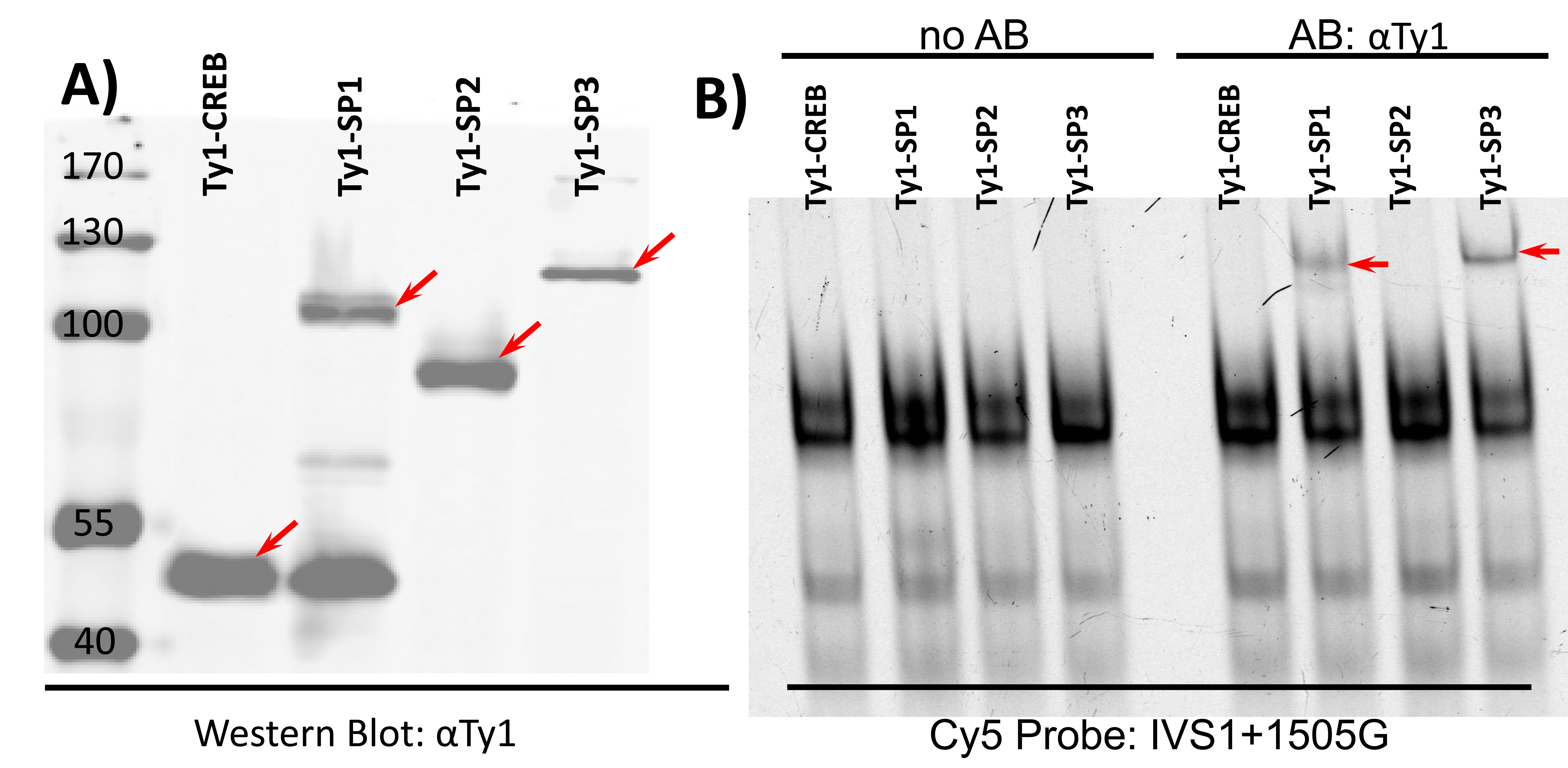

Supplement: Figure S6 — Epitope tagged versions of SP1 and SP3, but not CREB and SP2 bind to the IVS1+1505G probe. Immortalised brown adipocytes were infected with retrovirus expressing the full lenght cDNA of either CREB, SP1, SP2 or SP3 that were fused to a 2x Ty1 Tag at their N-terminus. Cells were used to generate RIPA extracts for a Western Blot (A) and nuclear extracts to perform EMSA supershift experiments (B). Red arrows denote the specific signals/supershift. (TIF) [file pone.0083426.s006.tif]

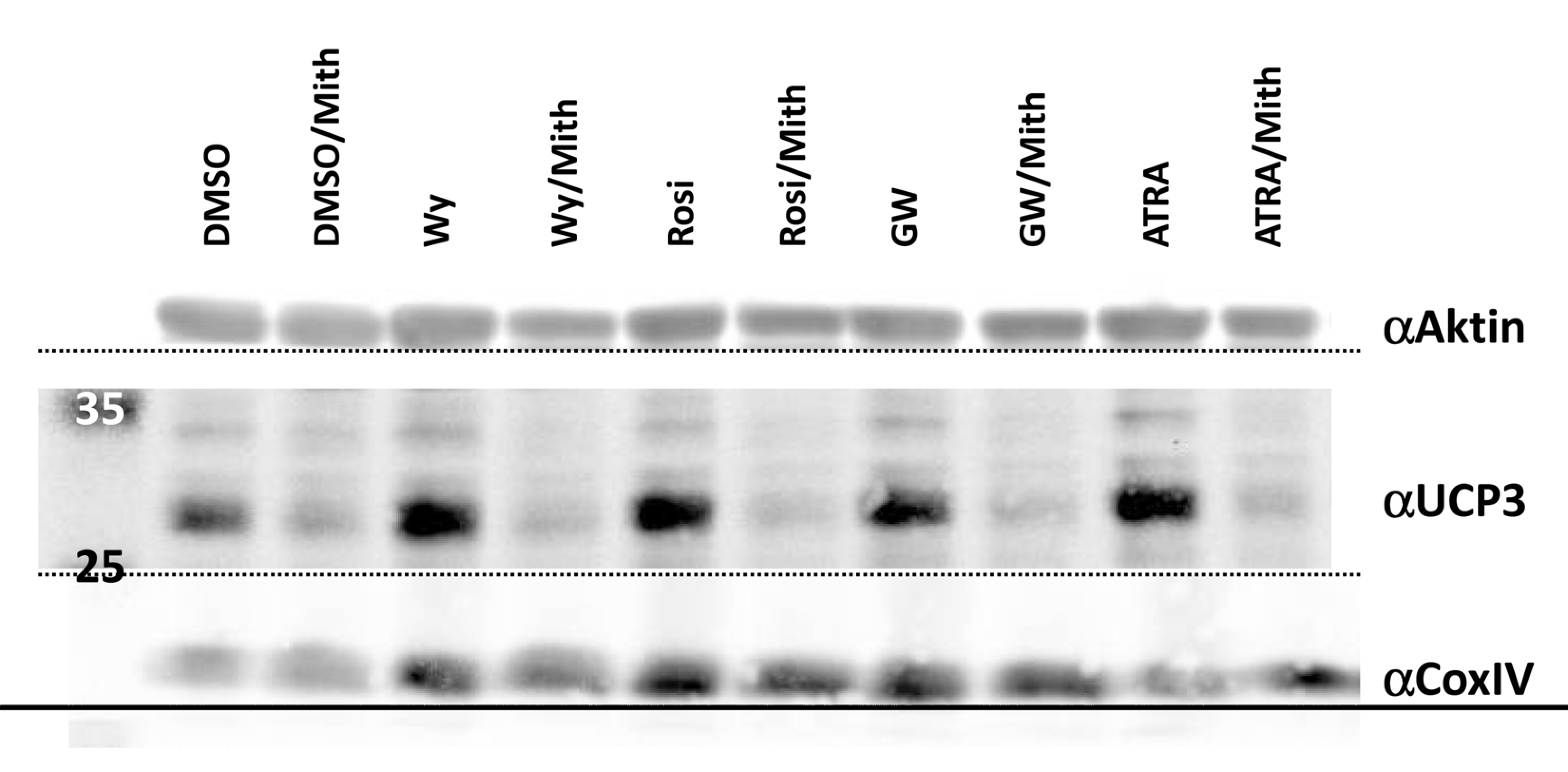

Supplement: Figure S7 — Mithramycin treatment decreases abundance of UCP3 Protein in immortalised brown adipocytes. Immortalised brown preadipocytes were induced and differentiated until full differentiation and and treated with Wy14643 (5 µM, PPARα agonist), Rosiglitazone (5 µM, PPARγ agonist), GW0742 (0,4 µM, PPARδ agonist) and All-Trans-Retinoic Acid (5 µM, RXR/RAR agonist) in presence or absence of 0,4 µM Mithramycin for 30 hours. RIPA extracts were generated and a Western blot against UCP3 (Pierce PA1-055), panAktin and CoxIV was performed. 40 µg protein were loaded per lane. Dottet lines indicate that the membrane was cut into 3 pieces. (TIF) [file pone.0083426.s007.tif]
