# Supplementary material for: A Novel SP1/SP3 Dependent Intronic Enhancer Governing Transcription of the UCP3 Gene in Brown Adipocytes
Source: PLoS One. 2013 Dec 31;8(12):e83426. doi: 10.1371/journal.pone.0083426 (PMC3877035; doi:10.1371/journal.pone.0083426)
Supplement: Table S1 — Oligonucleotides used for deletions/mutagenesis. (DOC) [file pone.0083426.s008.doc]

**Table S1:** Oligonucleotides used for deletions/mutagenesis.

| **Deletion** | **Region** | **primers used** |
| --- | --- | --- |
| **∆1 fw** | 3810-4129 | TGTGAGCCTCCATGAAAGGGAG |
| **∆1 rev** | 3810-4129 | TTGCAAAGGTCTGCTGCCCA |
| **∆2 fw** | 4171-4483 | ACTGTTGCTTGAGGAAGCCTGG |
| **∆2 rev** | 4171-4483 | GCCCTGAGGTCATGTCTGACCTTA |
| **∆4a fw** | 4850-5155 | CCAGAGCACTTTCTGCTGGAGG |
| **∆4a rev** | 4850-5155 | CCAGTACCTCCTGCTGGGAAGG |
| **∆4b fw** | 4850-5191 | CCAGAGCACTTTCTGCTGGAGG |
| **∆4b rev** | 4850-5191 | GCCCTTCTGTAGGGCAAAGGG |
| **∆5 fw** | 5199-5299 | AGAAGTAGTATAGTCCCCTTTGCCCTACAGAAGG |
| **∆5 rev** | 5199-5299 | AGAAGTAGTATTAACACGCCTGCACTGTTGGTAC |
| **∆6 fw** | 5299-5719 | CTGGGTACCAACAGTGCAGGC |
| **∆6 rev** | 5299-5719 | CCAAAGTGAGTTTGAATCTAAAAGAGGCTG |
| **∆7 fw** | 5715-5947 | GCAGGGTAGTTTTTAAATCTAGTTTACAGATGAGAAA |
| **∆7 rev** | 5715-5947 | CAACCTATATCAGATGTCCTGAATATCAGATATTTGTATT |
| **∆8 fw** | 6018-6273 | CACTGCAATTTTACCACTGTTATGAAGCAT |
| **∆8 rev** | 6018-6273 | TGTCTGTACTGACCAAGGTTCCGC |
| **∆9 fw** | 6273-6632 | GGAGGAGCTGTCAGGAAGGGAC |
| **∆9 rev** | 6273-6632 | GCCATGAGATGGATGAGAGTGATACTG |
| **∆10 fw** | 6631-6891 | CAGATACCCCTCGACCCTGTCTC |
| **∆10 rev** | 6631-6891 | CCCTGGAAATAGAACAGAGCCAGACA |
| **∆Int fw** | 3738-6955 | TGTGAGTCTAGCCAAGGTAGGGTATGC |
| **∆Int rev** | 3738-6955 | CTGCCCCCCGGAACTGAAGTA |
| **QC DRPro fw** | 3583-3597 | GTCAACTAGCTTCTCAGAATTGATATCGCTGGTGCGTAAGGCC |
| **QC DRPro rev** | 3583-3597 | GGCCTTACGCACCAGCGATATCAATTCTGAGAAGCTAGTTGAC |
| **QC DRInt fw** | 5203-5214 | GACCTGGCTCCCTTCTTCTGTAACTCGAGAAACTAAGGCCTGAATAAGTG |
| **QC DRInt rev** | 5203-5214 | CACTTATTCAGGCCTTAGTTTCTCGAGTTACAGAAGAAGGGAGCCAGGTC |

Base numbering according to AY523564.2
