# Supplementary material for: A Novel SP1/SP3 Dependent Intronic Enhancer Governing Transcription of the UCP3 Gene in Brown Adipocytes
Source: PLoS One. 2013 Dec 31;8(12):e83426. doi: 10.1371/journal.pone.0083426 (PMC3877035; doi:10.1371/journal.pone.0083426)
Supplement: Table S2 — miRNA sequences, top strand of 2 complementary oligonucleotides. (DOC) [file pone.0083426.s009.doc]

**Table S2:** miRNA sequences, top strand of 2 complementary oligonucleotides

| **miRNA** | **Top strand sequence according to sequencing reaction** |
| --- | --- |
| SP1 miR1 | TGCTGTTGAGCAGCATTCACAGTGACGTTTTGGCCACTGACTGACGTCACTGTATGCTGCTCAA |
| SP1 miR2 | TGCTGTCAAGCATCAGACTATACTTCGTTTTGGCCACTGACTGACGAAGTATACTGATGCTTGA |
| SP3 miR1 | TGCTGTTTGATTAGAGCCAGGAATGAGTTTTGGCCACTGACTGACTCATTCCTCTCTAATCAAA |
| Sp3 miR2 | TGCTGTAATCAAGGCATCATCTCTTCGTTTTGGCCACTGACTGACGAAGAGATTGCCTTGATTA |
| shBle miR1 | TGCTGTGATGAACAGGGTCACGTCGTGTTTTGGCCACTGACTGACACGACGTGCCTGTTCATCA |
| LacZ miR1 | TGCTGAAATGTACTGCGCGTGGAGACGTTTTGGCCACTGACTGACGTCTCCACGCAGTACATTT |
| UCP1 miR1 | TGCTGAAGAGAAGTACTCTTGGACTGGTTTTGGCCACTGACTGACCAGTCCAAGTACTTCTCTT |
| UCP1 miR2 | TGCTGTTATGTGGTACAATCCACTGTGTTTTGGCCACTGACTGACACAGTGGAGTACCACATAA |
