# Supplementary material for: A Novel SP1/SP3 Dependent Intronic Enhancer Governing Transcription of the UCP3 Gene in Brown Adipocytes
Source: PLoS One. 2013 Dec 31;8(12):e83426. doi: 10.1371/journal.pone.0083426 (PMC3877035; doi:10.1371/journal.pone.0083426)
Supplement: Table S3 — miRNA combinations in the different viral constructs. (DOC) [file pone.0083426.s010.doc]

**Table S3:** miRNA combinations in the different viral constructs

| **Virus construct** | **Contains combination of miRNAs** |
| --- | --- |
| SP1 single KD | SP1 miR1, SP1 miR2 |
| SP3 single KD | SP3 miR1, SP3 miR2 |
| SP1&SP3 double KD A | SP1 miR1, SP3 miR1 |
| SP1&SP3 double KD B | SP1 miR2, SP3 miR1 |
| Ctrl Z | LacZ miR1, shBle miR1 |
| Ctrl U | UCP1 miR1, UCP1 miR2 |
