# Supplementary material for: A Novel SP1/SP3 Dependent Intronic Enhancer Governing Transcription of the UCP3 Gene in Brown Adipocytes
Source: PLoS One. 2013 Dec 31;8(12):e83426. doi: 10.1371/journal.pone.0083426 (PMC3877035; doi:10.1371/journal.pone.0083426)
Supplement: Table S4 — Primers for amplification of the GFP+miR cassette for transfer into pMXs. (DOC) [file pone.0083426.s011.doc]

**Table S4:** Primer for amplification of the GFP+miR cassette for transfer into pMXs

| **Primers for amplification of the miRNA cassette** | |
| --- | --- |
| GFP+miR fw | AGAGGTCTCCGATCAGAGAACCCACTGCTTACTGGC |
| GFP+miR re | AGAGGTCTCGTCGAGGGCCCTCTAGATCAACCACTTT |
