# Supplementary material for: A Novel SP1/SP3 Dependent Intronic Enhancer Governing Transcription of the UCP3 Gene in Brown Adipocytes
Source: PLoS One. 2013 Dec 31;8(12):e83426. doi: 10.1371/journal.pone.0083426 (PMC3877035; doi:10.1371/journal.pone.0083426)
Supplement: Table S5 — shRNA sequences in pTER. (DOC) [file pone.0083426.s012.doc]

**Table S5:** shRNAs in pTER.

| **Pool scrambled shRNAs, top strand** | |
| --- | --- |
| scram1 | GAGGAGTTCGTGTTACTAATTCAAGAGATTAGTAACACGAACTCCTCTTTTT |
| scram2 | GACCTACTATATGTAACTATTCAAGAGATAGTTACATATAGTAGGTCTTTTT |
| scram3 | GCATGGACACGTCTAATAATTCAAGAGATTATTAGACGTGTCCATGCTTTTT |
| scram4 | GACACCTTGAGCTTATAGATTCAAGAGATCTATAAGCTCAAGGTGTCTTTTT |
| **Pool shRNAs targeting SP1, top strand** | |
| shSP1-1 | GGATGGATCTGGTCAAATATTCAAGAGATATTTGACCAGAACCATCCTTTTT |
| shSP1-2 | CATCATGCCTTGATAAATATTCAAGAGATATTTATCAAGGCATGATGTTTTT |
| shSP1-3 | GATCACTCCATGGATGAAATTCAAGAGATTTCATCCATGGAGTGATCTTTTT |
| shSP1-4 | GACTCAGTATGTGACCAATTTCAAGAGAATTGGTCACATACTGAGTCTTTTT |
| **Pool shRNAs targeting SP3, top strand** | |
| shSP3-1 | ATTATATCCAGTCGCCTGTTTCAAGAGAACAGGCGACTGGATATAATTTTTT |
| shSP3-2 | GCTATGGATAGTTCAGACATTCAAGAGATGTCTGAACTATCCATAGCTTTTT |
| shSP3-3 | GCAACACATTTGTCATATATTCAAGAGATATATGACAAATGTGTTGCTTTTT |
| shSP3-4 | GCCAGTGGTCAAAATATATTTCAAGAGAATATATTTTGACCACTGGCTTTTT |

Vectors were kindly provided by Dr. Guntram Suske
