# Supplementary material for: A Novel SP1/SP3 Dependent Intronic Enhancer Governing Transcription of the UCP3 Gene in Brown Adipocytes
Source: PLoS One. 2013 Dec 31;8(12):e83426. doi: 10.1371/journal.pone.0083426 (PMC3877035; doi:10.1371/journal.pone.0083426)
Supplement: Table S6 — Top strands of probes and competitors used in EMSA. (DOC) [file pone.0083426.s013.doc]

**Table S6:** Top strands of probes and competitors used in EMSA.

| **Name** | **Top strand sequence** |
| --- | --- |
| IVS1505G Cy5 | 5'Cy5-GTGTTTTCTTAACACGCCTGCACTGTTGGTA |
| IVS1505A Cy5 | 5'Cy5-GTGTTTTCTTAACACACCTGCACTGTTGGTA |
| SP1 consensus Cy5 | 5'Cy5-ATTCGATCGGGGCGGGGCGAGC |
| IVS1505G | GTGTTTTCTTAACACGCCTGCACTGTTGGTA |
| IVS1505A | GTGTTTTCTTAACACACCTGCACTGTTGGTA |
| SP1 consensus | ATTCGATCGGGGCGGGGCGAGC |
| IVS1504A | GTGTTTTCTTAACAAGCCTGCACTGTTGGTA |
| Pig putative GC Box 1 | GTGCCCACCTAGGGGCAGGGAGGGAGTTCAG |
| Pig putative GC Box 2 | AGGGAGTTCAGTCCCTCCACCAAGGCTGACC |
| Human putative GC Box | TGAAGTTCCTGGGGGCAGGCACAGCAGCCTG |
| Mouse putative GC Box | CATGTCTCTAAACATGCCTACCCTGCTCTTC |
| Rat putative GC Box | TTCTCTAAACACGCCCACACCGCTA |
| AP1 | TTGTCCTAGTCAGCCAGCTGTG |
| C/ebp | GTGTGTATTATGCAGGACTGCACTGTTGGTA |
| Cdxa | GCATTTTATTACCACGCCTGCACTGTTGGTA |
| Cmyb | GTGTTTTCAGCAACCGCCTGCACTGTTGGTA |
| CTCF | TGCTTGAGTGCCCTCTGGTGGGCAATAGGA |
| ETF | GTGTTTTCACATTCCTCCGGCACTGTTGGTA |
| Msx | GTGTTTTTCTAATTGGCCTGCACTGTTGGTA |
| MyoD | CCCCCAACAGCTGTTGCCTGA |
| NFκB | TTGGCTGTGGTACTTTCCAACGGAA |
| p53 | GTGTTTCAGGACATGTCCAGGCATGTCTCTA |
| Pbx | GTGGATGATTGACAGGTCTGCACTGTTGGTA |
| Sry | GTGTTTTGTAAACAATAGTGCACTGTTGGTA |
| Tbp | GTGTTTTGGTATAAATCCTGCACTGTTGGTA |

All oligos were annealed with their respective unlabeled complement.
