# Supplementary material for: A Novel SP1/SP3 Dependent Intronic Enhancer Governing Transcription of the UCP3 Gene in Brown Adipocytes
Source: PLoS One. 2013 Dec 31;8(12):e83426. doi: 10.1371/journal.pone.0083426 (PMC3877035; doi:10.1371/journal.pone.0083426)
Supplement: Table S7 — Sequencing primers used for validation of constructs. (DOC) [file pone.0083426.s014.doc]

**Table S7:** Sequencing primers used for validation of constructs

| **Sequencing primers** | |
| --- | --- |
| pMXs fw seq | CTTACACAGTCCTGCTGACCAC |
| pMXs re seq | ACATATAGACAAACGCACACCG |
| BlockIt fw seq | GACAACCACTACCTGAGCAC |
| pGL3 re seq (Luciferase) | GGTTTGTCCAAACTCATCAATG |
| SP/DR fw seq | CCTAGCTCTCCAGGCAAATC |
| SP/DR re seq | TCTCCAGGCTCTTCCTTCAG |
| pGL3 fw seq (Promoter) | CTAGCAAAATAGGCTGTCCC |
| pTer re seq | ACTAGAAGGCACAGTCGAGG |
