# Supplementary material for: A Novel SP1/SP3 Dependent Intronic Enhancer Governing Transcription of the UCP3 Gene in Brown Adipocytes
Source: PLoS One. 2013 Dec 31;8(12):e83426. doi: 10.1371/journal.pone.0083426 (PMC3877035; doi:10.1371/journal.pone.0083426)
Supplement: Table S8 — Oligonucleotides for construction of tagged overexpression Vectors. (DOC) [file pone.0083426.s015.doc]

| 2x Ty1 for | GATCGCCACCATGGGAgaggtgcacaccaaccaggaccccctggacg  ccgaggtgcacaccaaccaggaccccctggacgccGGATCCCAAGC |
| --- | --- |
| 2x Ty1 rev | GGCCGCTTGGGATCCggcgtccagggggtcctggttggtgtgcacct  cggcgtccagggggtcctggttggtgtgcacctcTCCCATGGTGGC |
| CREB tag for | CGTCTCGGATCCATGACCATGGAATCTGGAGC |
| CREB tag rev | CGTCTCCGGCCTCAATCTGATTTGTGGCAGTAAAGG |
| SP1 tag for | CGTCTCGGATCCATGAGCGACCAAGATCACTCC |
| SP1 tag rev | CGTCTCCGGCCAGATGTCTCTTGGACCCA |
| SP2 tag for | GGATCCATGAGCGCAGATCCACAGAT |
| SP2 tag rev | GCGGCCGCCTCAGTTGGCCTTACAAGC |
| SP3 tag for | CGTCTCGGATCCATGACCGCTCCCGAAAAGC |
| SP3 tag rev | CGTCTCCGGCCTTACTCCATTGTCTCATTTCCAGA |
