# Supplementary material for: A Novel SP1/SP3 Dependent Intronic Enhancer Governing Transcription of the UCP3 Gene in Brown Adipocytes
Source: PLoS One. 2013 Dec 31;8(12):e83426. doi: 10.1371/journal.pone.0083426 (PMC3877035; doi:10.1371/journal.pone.0083426)
Supplement: Method S1 — Human sequence variations. (DOCX) [file pone.0083426.s016.docx]

Supplement:

Bi-directional Sanger re-sequencing (primers: UCP3 F 5’-CCCTCCTGGGTAAGGAAGAG-3’; UCP3 R 5’-ACTAGCCCCTCCTTCCATGT-3’; chr11: 73,717,911 - 73,718,260, 350 bps) of the homologous *UCP3* regulatory element (chr11: 73,718,021 - 73,718,037) in 95 extremely obese children and adolescents and 96 underweight adult controls did not reveal a sequence variation in the DR SP element region. A non-synonymous SNP in *UCP3* 33 bps downstream of the regulatory element (rs8179180, Val9Met) was the only variant detected in the analyzed fragment. The minor allele frequencies were low in both cases (0.53%) and controls (1%).
